# The increasing complexity of arbovirus serology: An in-depth systematic review on cross-reactivity

**DOI:** 10.1371/journal.pntd.0011651

**Published:** 2023-09-22

**Authors:** Louella M. R. Kasbergen, David F. Nieuwenhuijse, Erwin de Bruin, Reina S. Sikkema, Marion P. G. Koopmans

**Affiliations:** Department of Viroscience, Erasmus University Medical Center, Rotterdam, The Netherlands; University of Texas Medical Branch, UNITED STATES

## Abstract

Diagnosis of arbovirus infection or exposure by antibody testing is becoming increasingly difficult due to global expansion of arboviruses, which induce antibodies that may (cross-)react in serological assays. We provide a systematic review of the current knowledge and knowledge gaps in differential arbovirus serology. The search included Medline, Embase and Web of Science databases and identified 911 publications which were reduced to 102 after exclusion of studies not providing data on possible cross-reactivity or studies that did not meet the inclusion criteria regarding confirmation of virus exposure of reference population sets. Using a scoring system to further assess quality of studies, we show that the majority of the selected papers (N = 102) provides insufficient detail to support conclusions on specificity of serological outcomes with regards to elucidating antibody cross-reactivity. Along with the lack of standardization of assays, metadata such as time of illness onset, vaccination, infection and travel history, age and specificity of serological methods were most frequently missing. Given the critical role of serology for diagnosis and surveillance of arbovirus infections, better standards for reporting, as well as the development of more (standardized) specific serological assays that allow discrimination between exposures to multiple different arboviruses, are a large global unmet need.

## Author summary

Arboviruses are notorious for triggering antibodies that may cross-react with (closely) related arboviruses, complicating interpretation of serology-based arbovirus diagnostics and of epidemiological surveys. This is further complicated by the rapid global spread of arboviruses, that may lead to increasing co-circulation of antigenically related arboviruses, and by the lack of detailed background information on the population tested, methods used and background arbovirus exposures such as prior infections or vaccinations.

Here, we aim to provide a better understanding of the complex cross-reactivity patterns as well as the current knowledge and knowledge gaps in differential arbovirus serology. We have done a systematic analysis of reported cross-reactivity patterns from published peer-reviewed articles, using a system that allows assessment of the quality of reported cross-reactivity results. We show that using this approach, the majority of studies lack details needed to reliably assess whether reactivity to antigens from different, related arboviruses is caused by antibody cross-reactivity, or if it reflects antibodies from multiple exposures to these different viruses. Furthermore, there is a strong need for standardization of assays and study designs in future serological studies to compare cross-reactivity results from different studies. Tackling this may lead to improved diagnosis and surveillance of (newly) emerging and/or rapidly spreading arboviruses.

## Introduction

In the past two decades, the global impact of arthropod-borne (ARBO) viruses has steadily been increasing due to rapid distribution over larger geographic areas, the emergence of new variants, and new complications arising from the sequel of viral exposures potentially leading to enhanced disease [1–3]. Major anthropogenic factors contributing to this global spread are increased human travel to or from (sub)tropical arbovirus endemic regions, global transportation of water-retaining objects offering opportunity for dispersal of mosquitos, urbanization and deforestation. Climate change may also have an impact by influencing the geographical and temporal distribution of arthropods and/or reservoir hosts such as migrating birds [3–7]. Recent examples of unusual arboviral spread are autochthonous chikungunya virus (CHIKV) cases that have been reported in Italy and France throughout the past years [8–12], Usutu virus (USUV) which is frequently observed in birds and occasionally in humans in Europe [13], the Zika virus (ZIKV) epidemic that rapidly spread to the Pacific islands and the Americas [14–20], Crimean Congo Haemorrhagic fever virus (CCHFV) expansion in Europe [6,21–23], the first autochthonous West Nile virus (WNV) cases in Germany [24] and the Netherlands [25], and locally acquired dengue virus (DENV) cases in France and Spain [26]. Furthermore, there are indications that other arboviruses such as Mayaro virus (MAYV) are further spreading into the Caribbean and Central and South America, likely causing more outbreaks in the near future [27–32].

As clinical symptoms of most arboviruses are very similar and virological (molecular) detection is only possible in a short period after clinical symptoms due to the short arbovirus viremic phase, serology is essential in arboviral diagnosis. However, serology-based differential arbovirus diagnosis is complicated by antibodies induced by a primary arbovirus infection that may cross-react with other closely related arboviruses. This is further complicated by the phenomenon original antigenic sin (OAS), which describes the preferential boosting of these cross-reactive antibodies upon subsequent heterologous arbovirus infections [33]. Together with the current global spread of arboviruses, which increasingly co-circulate [34–38], individuals living in arbovirus endemic areas progressively carry cross-reactive antibodies from previous exposures with increasing age [39]. For instance, even though efforts have been made to develop assays with increased specificity [40–43], ZIKV and DENV antibodies highly cross-react in DENV endemic settings [39,44–46]. While serological cross-reactivity between ZIKV and DENV is expected given that they are closely related, much less is known about cross-reactive antibody patterns of arboviruses other than ZIKV or DENV. This also applies to cross-reactive antibodies that may be induced by arbovirus vaccination. Given the increased arbovirus spread and co-circulation, there is increasing need for elucidating cross-reactive antibody responses of less studied but prevalent arboviruses in the context of differential diagnosis.

Besides the diagnostic challenge, understanding patterns of cross-reactivity is also important because cross-reactive pre-existing heterotypic arbovirus antibodies can potentially enhance infection of a heterologous virus strain via antibody dependent enhancement (ADE), as seen between different DENV serotypes, which may exacerbate disease [2,47–60]. Therefore, boosting these cross-reactive antibodies by sequential heterologous arbovirus infections, may result in higher chances of developing severe disease by ADE. This was recently also confirmed in the wake of the Zika virus epidemic demonstrating elevated risk of DENV severe disease by either a prior ZIKV or DENV infection or both [61]. Moreover, the safety issues seen with Dengvaxia vaccination; where younger-aged baseline DENV seronegative recipients experienced enhanced disease approximately 18 months after vaccination, might be explained by ADE as well [62].The current hypothesis is that Dengvaxia mimics a primary infection in these DENV seronegative individuals, putting the recipients at risk of ADE and developing severe disease when experiencing a subsequent DENV infection [62]. However, when it comes to enhancement of ZIKV infection due to prior DENV infection, there is contrasting evidence [63]. This possibly might be explained by the DENV antibody levels and length of the time interval between the sequential heterologous infections being crucial in determining whether it will lead to protection or enhancement of ZIKV infection [63]. Although ADE and the risk of developing severe disease has been demonstrated for ZIKV and DENV, it remains to be determined whether this also plays a role for other arboviruses [64].

The problems of serological differential diagnosis and the potential risk of antibody-mediated enhanced disease highlight the importance of improving insight into the complex antibody reactivities following exposure to one or more (neglected) arboviruses. A challenge, however, is that information that can be used to accurately interpret serological test results, such as detailed background information on the population tested, methods used, and information on background exposures to other arboviruses often are not available or provided, thus hampering proper interpretation of study results. Therefore, in this review, we have done a systematic analysis of reported multi-antigen antibody-reactivity patterns from published peer-reviewed articles, using a system that allows scoring risk of bias and thereby assessment of the quality of reported results to be able to interpret antibody cross-reactivity. To do this, we reviewed papers for completeness of information on geographic region, travel history, vaccination history, infection history, age group, case definitions used, timing of sampling, specification of serological methods, confirmation of infection and study size. This information was subsequently used to determine possible arbovirus background exposure(s) and reliability of the final diagnosis to estimate whether the results reflect antibody specific- or cross-reactivity.

## Materials and methods

### Search strategy and selection criteria

**Fig 1:**
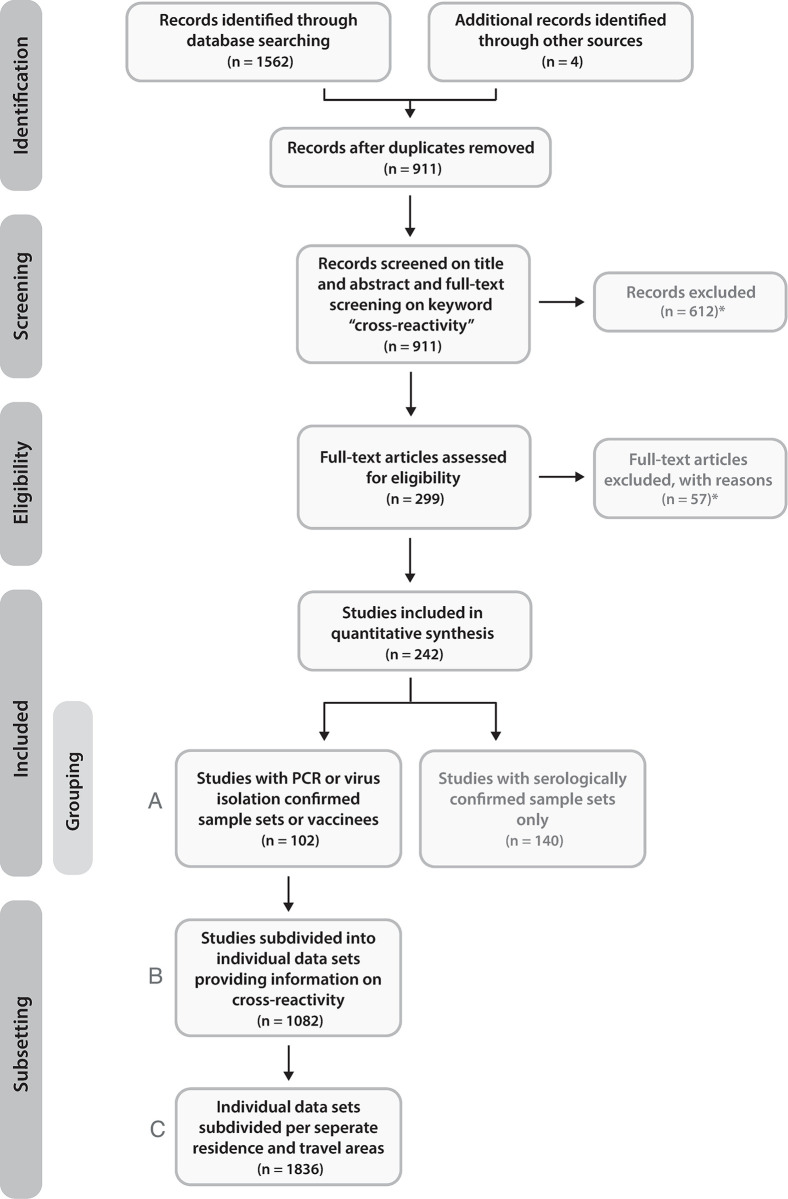
Overview of article selection in literature search. Overview of the selection process of articles that study arbovirus antibody cross-reactivity in humans. Only studies with at least one vaccinee study group or one or more PCR or virus isolation confirmed sample sets were used in this systematic review (N = 102) (A). Studies were subdivided into 1082 individual data sets with different characteristics to assess cross-reactivity (B). Individual data sets were further subdivided to illustrate different residence and travel areas of included studies (N = 1836) (C). *Reasons for exclusion were not studying arbovirus cross-reactivity in humans and/or not describing serological cross-reactivity in sufficient detail (e.g., describing the specific viruses assessed for cross-reactive binding).

We searched Medline, Embase and Web of Science for serological studies on arthropod-borne Flavi-, Toga-, Bunya-, and Reoviruses using a search strategy specifically designed to capture studies addressing cross-reactivity of antibodies (Table A in S1 Appendix). The total period covered was all published articles up to and including March 2023. Reviews, meta-analyses, case-reports and animal studies were excluded from the search (Table A in S1 Appendix). The search resulted in a total of 1562 articles, and together with four additional studies found separately from the search by PubMed, this amounted up to 911 studies (Fig 1). All articles were first screened by title and abstract and subsequently by full text, excluding 669 articles not describing serological cross-reactivity of arboviruses in humans or not in sufficient detail (e.g., missing information regarding the specific viruses studied for cross-reactivity) (Fig 1). The remaining 242 studies were screened for the level of evidence of exposure; only studies in which at least part of the participants were vaccinees or confirmed positive by molecular detection or virus isolation were included, since in these cases the causative agent is confirmed [65–69]. The final selection consisted of 102 articles (Fig 1A and Table B in S1 Appendix and S1 Datafile). Subsequently, each study that described measured arbovirus cross-reactivity was subdivided into individual data sets based on different specifics (N = 1082), such as different methods used to diagnose patients and/or measure the amount of cross-reactivity, as well as using different case definitions, sampling time, residence and/or travel areas, age, vaccination status, infection history and study size (Fig 1B). To illustrate all different residence areas and/or travel areas of the 1082 individual data sets, the data was further subdivided where needed, even if cross-reactivity results of the data set were not presented per separate residence or travel area of the studied patients or vaccinees. This amounted to 1836 residence and travel areas on the level of country (Fig 1C). Figures were made using R software (S1 Datafile).

### Reliability scoring system

**Fig 2:**
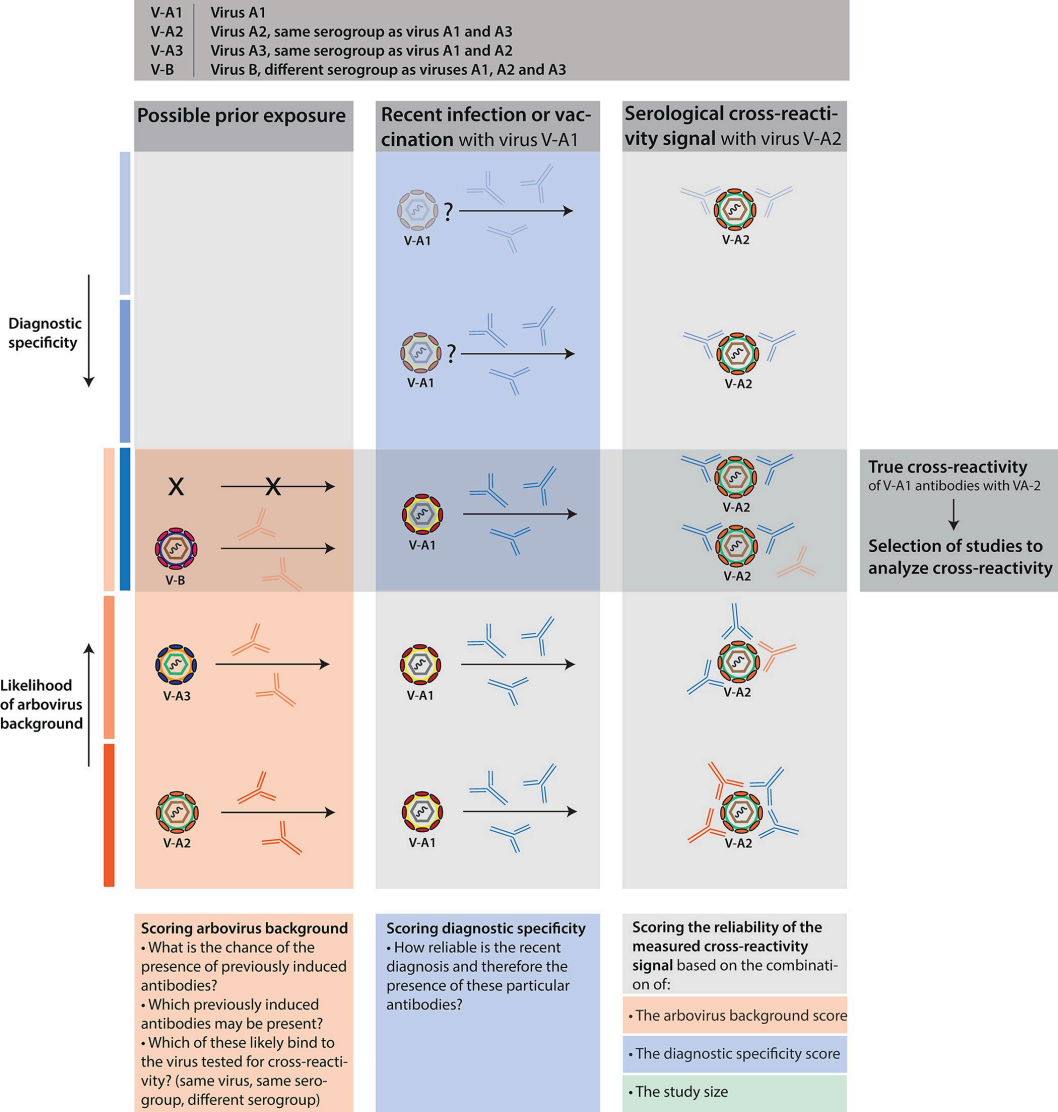
Principle of scoring the reliability of cross-reactivity signals. True cross-reactivity is defined as the combination of a high diagnostic certainty (diagnostic specificity score), a low chance of bias by possible previous arbovirus exposures (arbovirus background score) together with a large study size (study size score). Study data sets considered reliable regarding the serological cross-reactivity signals were selected for analyzing cross-reactivity.

We aim to gain insight into the reliability of reported serological test results for diagnostic interpretation, for interpretation of seroepidemiological surveys, and for the assessment of antigenic cross-reactivity between arboviruses. To achieve this, we developed a reliability scoring system (Table 1), scoring risk of bias of the final serological result by combining a diagnostic specificity score, an arbovirus background score and a weight of the studies by study size (Table 1 and Fig 2). For each category, variables were either based on WHO and CDC recommendations and literature or defined by the study team (for details, see Supplemental Information A and Table C in S1 Appendix). Information provided on the timing of sampling, case definitions and the choice of assays was used for the diagnostic specificity score to estimate the probability of a correct diagnosis [65–69]. The arbovirus background score used knowledge on endemicity of different geographic regions for arboviruses listed in Tables D and E in S1 Appendix, as well as age, vaccination status and the prior infection history to assess the probability of presence of antibodies that may bias the serological results [65–69]. The rationale of scoring each variable was based on WHO and CDC recommendations, literature and expert input. For detailed information about the rationale of the scoring system and scoring variables, see Supplemental Information A in S1 Appendix. To equally weigh the three categories, scores were divided into quartiles for each category (Table F in S1 Appendix) and then combined into 5 final composite scores from highest quality (Group 1), yielding most conclusive information, to lowest quality studies (Group 5) in which conclusions regarding specificity of responses cannot be drawn with confidence (Tables G and H in S1 Appendix). This reliability system solely scores the level of antibody (cross-)reactivity interpretation, and does not score the overall quality of study results. For all scoring results of the individual data sets, see S1 Datafile. The presence of possible cross-reactive antibodies was calculated for each study and for each virus included in the multi-antigen serology panels. The calculated cross-reactivity percentages were divided into three groups: option 1) papers that described antibody reactivity in patients with (PCR) confirmed viral infection against both the infecting virus and a related virus in the same assay and/or the same antibody type (e.g., PCR confirmed ZIKV cases for which IgM reactivity was measured against both ZIKV and DENV using the same assay). Option 2) as 1, but where the serology for the heterologous viruses was done using a different assay and/or antibody type (e.g., PCR confirmed ZIKV cases that were also positive for ZIKV neutralizing antibodies, and for which IgM reactivity was measured against DENV, and option 3) papers describing antibody reactivity only for the heterologous antigen (e.g., PCR confirmed ZIKV cases for which IgM reactivity was measured against DENV, but ZIKV antibody reactivity was not tested or reported). Option 1 reflects the most accurate presentation of antibody cross-reactivity. All figures were made using R software.

**Table 1:** Reliability scoring system categories and variables used for scoring. Principles of scoring are based on either WHO or CDC recommendations [65–69], literature or were defined by the study team. For details see Supplemental Information A in *S1 Appendix*.

1 –Diagnostic specificity score	2 –Arbovirus background score	3 –Study size score
***Explanation*: *variables in this score provide information on the reliability of a positive or negative test result*, *assessing parameters needed for proper diagnostic interpretation*** *(variables b*, *c*, *d and e were only scored in study sets using serology as diagnostic method)*	***Explanation*: *variables used to form this composite score describe the quality of information about possible prior arbovirus exposures until time of sample collection***	***Explanation*: this score provides a weight based on study size.**
**a. Type of test and confirmation;** probability of correctly defined study groups by assessing type of (infection) confirmation: vaccinees, PCR or virus isolation and/or serology used	**a. Residence area (Tables C and D in** S1 **Appendix);** probability of bias by previous exposure to arboviruses circulating in the residence area. Bias probability is based on overlap in virus family and serogroup of the virus tested for cross-reactivity and possible prior exposure circulating viruses (same virus or unknown residence area, same serogroup, different serogroup)	**a. Study size;** probability of overall reliable cross-reactivity results by study size of groups used (unknown, 1–10, 11–100, 101–200, 201–1000)
**b. Serological case definition used;** paired or single sera, above cut-off only, seroconversion or 4-fold increase between samples and 4-fold difference with or lower/negative result for virus that likely cross-reacts.	**b. Travel history (Tables C and D in** S1 **Appendix);** as above but for travellers	
**c. Sera sampling timepoints;** probability of reliable serological results by the timing of sampling of sera used (single serum after 10 days post symptom onset (dpso) or paired sera before 7 dpso and after 14 dpso in all or part of samples, other timepoints or unknown)	**c. Age;** likelihood of previous arbovirus infections (children, children and adults, adults or unknown)	
**d. Type of serology method(s);** type of serology assay(s) (ELISA/IFA/HI/rapid test confirmed by VNT, VNT only, ELISA/IFA/HI/rapid test only, based on symptoms or unknown) used to confirm the patient’s infection to assess likelihood of correct infection confirmation	**d. Arbovirus vaccination history;** likelihood of bias is determined based on overlap in virus family and serogroup between virus tested for cross-reactivity and virus of previous vaccination (same virus or unknown arbovirus vaccination history, same serogroup, different serogroup, no previous vaccination with some or all possible arbovirus vaccines)	
**e. Serological method specificity;** probability of reliable outcome by method characteristics regarding type of antigens used (more-specific or cross-reactive antigens, whole virus, unknown) and additional steps to increase specificity (blocking, inhibition).	**e. Arbovirus infection history;** serological or molecular evidence of prior arbovirus infection (arbovirus-naive/primary infection in all individuals or part of them, arbovirus experienced/ secondary infection or unknown)	

### Role of the funding source

The funder of the study had no role in study design, data collection, data analysis, data interpretation, or writing of the report.

## Results

### Search overview output

**Fig 3:**
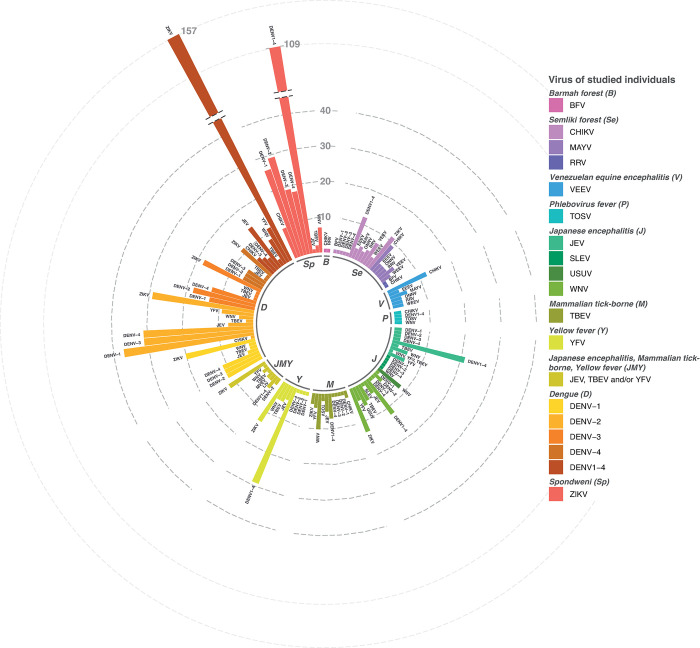
Overview of studies providing information on multi-antigen antibody-reactivity testing. Overview of all the combinations for which antibody measurements were done. Colours depict what the exposure had been for individuals sampled for serology testing (specified in the legend and the inner circle of the figure, ordered by serogroup), and the labels above each bar describe the virus antigens included in (cross-)reactivity panels. Total N is 1082 studies. Barmah forest (B), Semliki Forest (Se), Venezuelan equine encephalitis (V), Phlebovirus fever (P), Japanese encephalitis (J), Mammalian tick-borne (M), Yellow fever (Y), Japanese encephalitis, Mammalian tick-borne, Yellow Fever (JMY), Dengue (D), Spondweni (Sp).

The final selection included 102 publications describing arbovirus antibody reactivity to multiple antigens for vaccinees or patients (partly) confirmed by molecular detection and/or virus isolation (Fig 1 and Table B in S1 Appendix). Most articles used multiple patient groups infected with different arboviruses or from different areas in combination with different study designs and/or methods for virus identification or multi-antigen antibody measurements. Therefore, all separate patient and/or vaccinee groups were analysed as separate studies, amounting to a total of 1082 studies. Most studies were focused on DENV (514/1082; 47,5%), ZIKV (220/1082; 20,3%), Yellow fever virus (YFV) (60/1082; 5,5%) or CHIKV (52/1082; 4,8%) exposed individuals, whereas studies assessing multi-antigen antibody-reactivity for persons exposed to USUV (8/1082; 0,7%), Toscana virus (TOSV) (8/1082; 0,7%), Saint Louis encephalitis virus (SLEV) (5/1082; 0,5%), Ross River virus (RRV) (2/1082; 0,2%) or Barmah Forest virus (BFV) (2/1082; 0,2%) were sparse (Fig 3). The same pattern can be observed when looking at data on potential cross-reactivity; the majority of studies in this context was focused on ZIKV and DENV cross-reactivity (639 out of 1082; 59,1%), studying ZIKV-infection-induced antibody cross-reactivity with DENV (201 out of 639), DENV-infection-induced cross-reactive antibodies with ZIKV (235 out of 639) or DENV-infection-induced antibody cross-reactivity with a different DENV subtype (203 out of 639) (Fig 3). The remaining studies were studying serum panels collected after exposure to a wide range of arboviruses for cross-reactivity with arboviruses often in the same genus and/or serogroup as the virus of exposure (443 out of 1082, 40,9%) (Fig 3). Of these studies, the presence of DENV cross-reactive antibodies in sera collected after exposure to YFV (N = 33) and Japanese encephalitis virus (JEV) (N = 29) was most frequently studied (Fig 3). Out of all studies, cross-reactivity towards DENV was most often studied (523 out of 1082, 48,3%).

**Fig 4:**
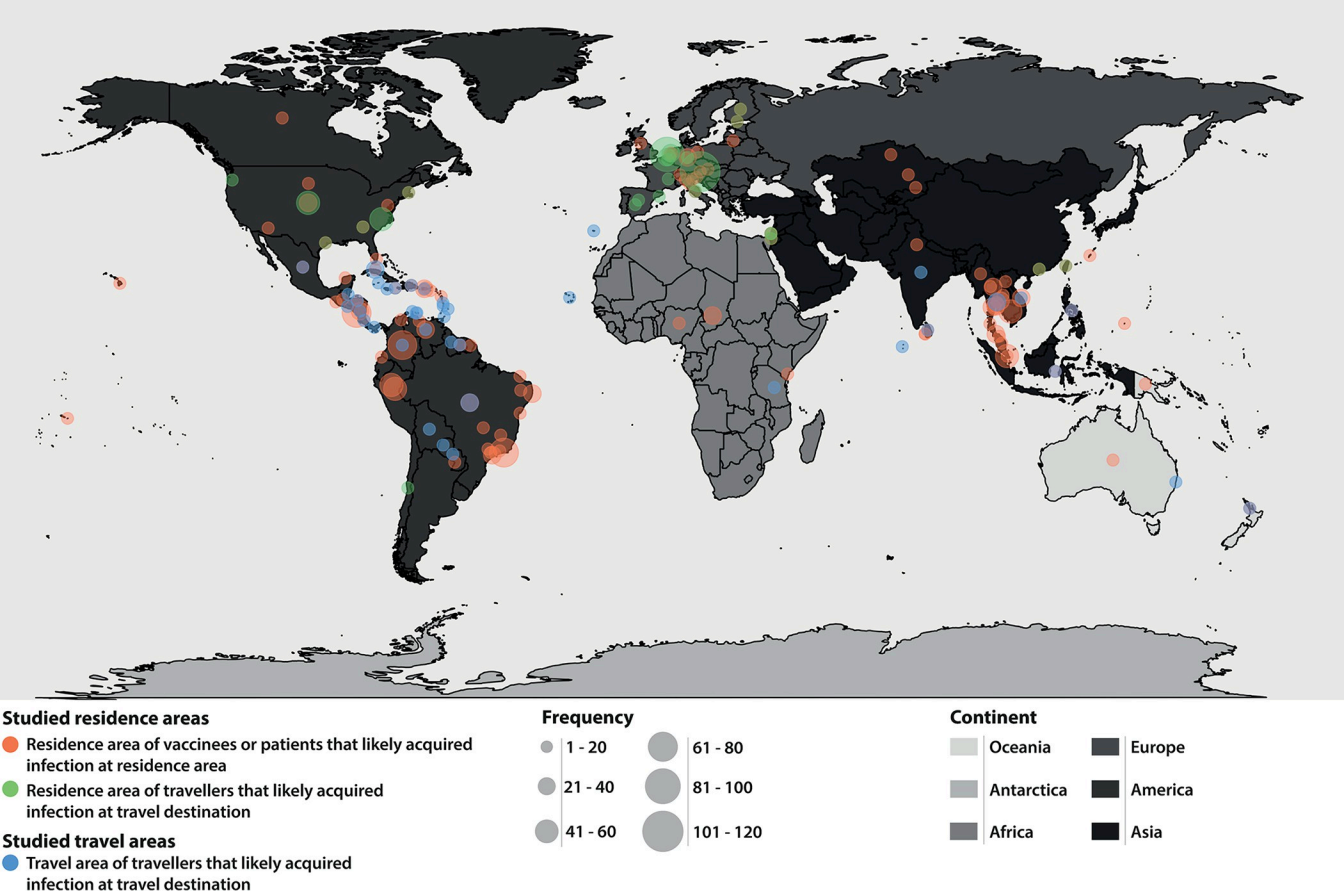
Overview of the geographic distribution of residence areas and travel destinations. Overview of all residence areas of studied individuals and travel destinations of travellers of the included studies (N = 1836). Residence areas of vaccinees or patients that did not have any (reported) prior travel and therefore (likely) acquired infection at the residence area are depicted in red (N = 1383). For travellers that likely got infected at the travel destination, residence areas are shown in green (N = 453) and the travel destinations in blue (N = 453). Study frequency is depicted by size and the continents are coloured using a grey-scale. Figure is made using the R package maps and Natural Earth (Cultural base layer, medium scale, countries). The world map, is made using the world data base of the R package maps: https://www.rdocumentation.org/packages/maps/versions/3.4.0.

Most studies described persons living in or travelling to South-, Central- or North America, Europe or South-east Asia (Fig 4), which are all areas where arboviruses are known to co-circulate [70–74] (Table D in S1 Appendix). Serology in residents or travellers to other geographic areas such as Africa, West and Central Asia and Oceania is less well studied (Fig 4), although arbovirus circulation has frequently been seen in these areas in the past as well [70–74] (Table D in S1 Appendix), highlighting an important research gap. In addition, more studies addressed serology in resident populations (874/1082; 80,8%) than in travellers (111/1082; 10,3%) or both or unknown (97/1082, 8,9%) (S1 Datafile). Most published studies had targeted diagnostic panels, with South American studies mainly focusing on the Spondweni- (ZIKV), Dengue (DENV 1–4), Semliki forest (CHIKV, MAYV) and Venezuelan equine encephalitis (VEEV) serogroups, whereas the majority of studies performed in other regions had narrow serology panels mostly limited to arboviruses from only one or two particular serogroups (S1 Datafile), in line with the known prevalence of circulating arboviruses in each area [70–74] (Table D in S1 Appendix). As expected, when included in (differential) diagnostic testing in a specific area, the same pattern was observed on the selection of arboviruses for multi-antigen-reactivity testing (Table D in S1 Appendix and S1 Datafile).

### Scoring studies to assess quality of multi-antigen-serological reactivity measurements

**Fig 5:**
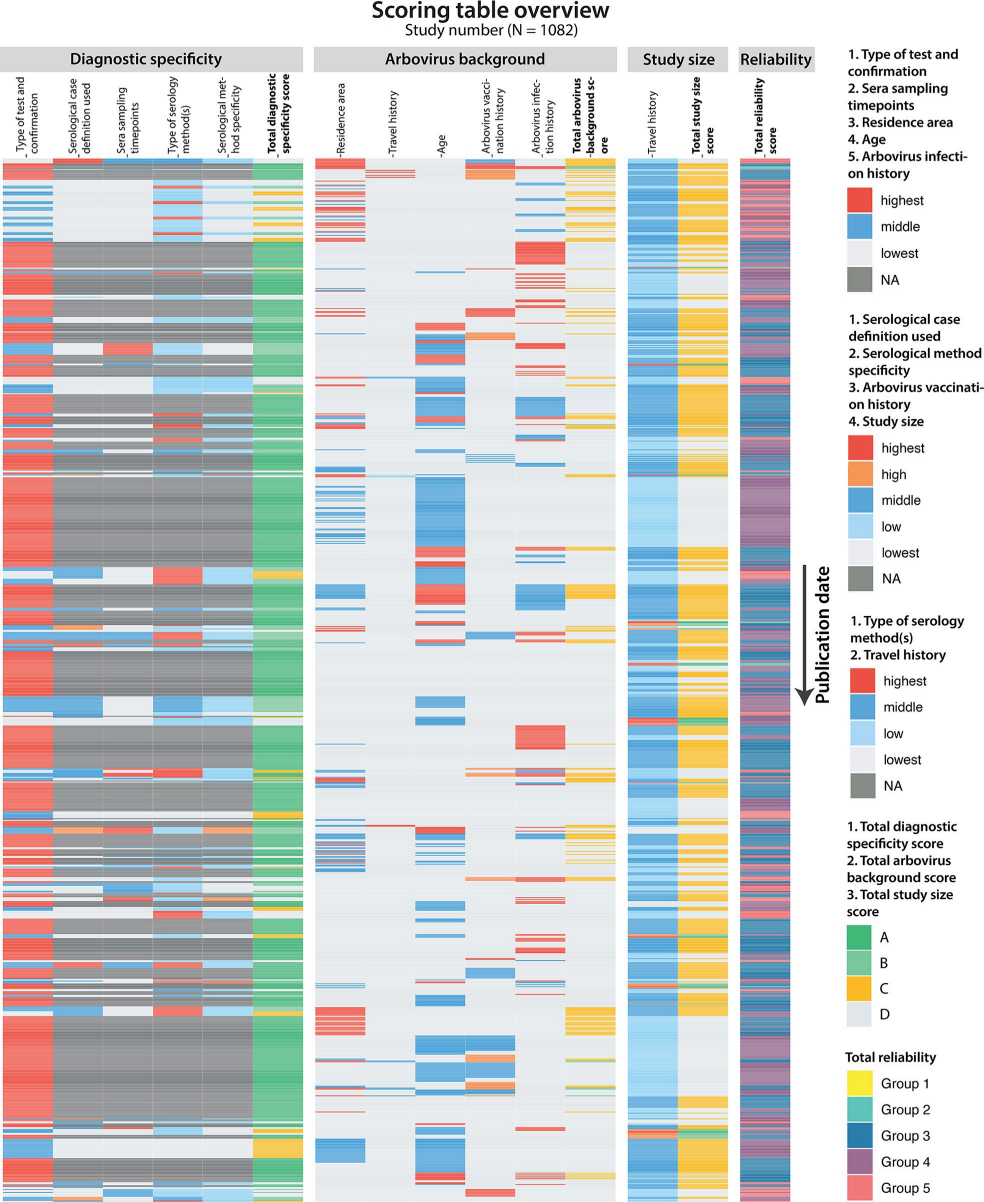
Overview of scoring results and total reliability of studies. Overview of scoring results of each category (diagnostic specificity, arbovirus background, study size) and total reliability for all included studies (N = 1082) ordered by publication date (oldest publication (left) to the most recent (right)). Scores of variables are divided into highest (red), high (orange), middle (dark blue), low (light blue) and lowest (light grey), whereby a high score indicates a low chance of bias and a low score a high chance of bias. The possible options for scoring can vary between variables (see legends per variable). Studies scoring highest on type of test and confirmation (studies with vaccinees or patients confirmed by PCR) are not evaluated for the other variables of the diagnostic specificity score weighing the serological diagnostic evidence (NA, dark grey). Total scores of each category are shown in four groups ranging from high to low: A (dark green), B (light green), C (yellow), D (light grey). The total composite reliability score of the studies is shown at the bottom ranging from highest (Group 1) to lowest (Group 5) in yellow, turquoise, dark blue, purple and pink, respectively.

**Fig 6:**
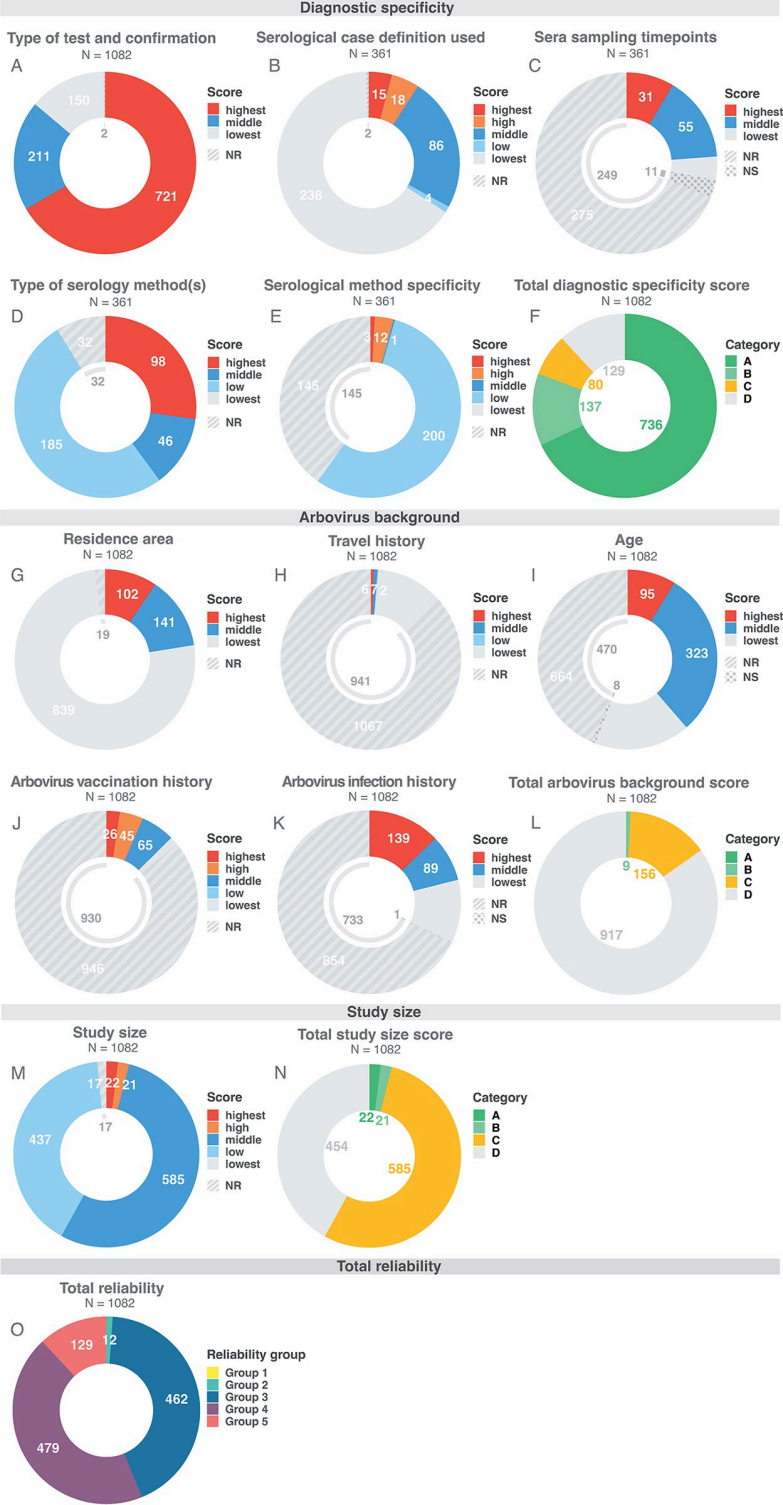
Distribution of (sub)category scores and total reliability groups. Scores per individual variable of each category and total reliability groups of all studies scored in the reliability scoring system. Color legend as in Fig 5. NR = information not reported (grey striped pattern), NS = information not sufficiently specified (grey dotted pattern). The number of studies (N) represents the number of subdivided data sets used to score each variable.

The results of scoring of the individual categories to assess the reliability of studies of antibody specificity are summarized in Figs 5 and 6 (for details, see Table C in S1 Appendix and S1 Datafile). Most studies involved persons who were vaccinated or had an arbovirus infection confirmed by RT-PCR or virus isolation (N = 721), and therefore had the highest score (A) for the category “diagnostic specificity” (Figs 5 and 6A and Table C in S1 Appendix). Studies using serology to define what the individuals had been exposed to rarely met the highest score (N = 15) due to low diagnostic quality or incomplete information regarding timing of sampling, case definitions and/or choice of method (specificity). A clear impact to the lower diagnostic specificity score was the limited number of studies reporting paired serum sample testing with high-quality based evidence (Figs 5 and 6B and Table C in S1 Appendix), which is considered important for reliable serological testing. Another, related, common issue was the timing of serum sampling; 76,2% of the studies had the lowest score possible (275/361, 76,2%), which was mainly due to the lack of this information (260/275, 94,5%) (Figs 5 and 6C and Table C in S1 Appendix). The type of serological methods used was mainly ELISA, IFA, HI or rapid diagnostic test only (185/361, 51,2%—low score), rather than the more specific serological testing using (confirmation) VNT assays (Figs 5 and 6D and Table C in S1 Appendix). Regarding (antigen) specificity of the serological methods: the most frequent category was the low score (200/361, 55,4%), and after that the lowest score as a result of missing details about method specificity (145/361, 40,2%) (Figs 5 and 6E and Table C in S1 Appendix). Despite the lower diagnostic specificity score for studies using serology with only 15 studies in the highest category (15/361, 4,2%—A), the second highest score did slightly have the highest frequency (137/361, 38%—B) (Figs 5 and 6F).

In order to assess the potential for serological (cross-)reactivity from exposure to other arboviruses, papers were scored for the risk of presence of (cross-)reacting antibodies from prior exposure as well as completeness of data provided on area of residence or travel, age and vaccination and infection history for an “arbovirus background score”. This data was largely missing, leading to the lowest possible score for most studies in this category. The only exception was reporting of the area of residence since only few studies did not report this information (19/1082, 1,8%). However, the vast majority received the lowest score (839/1082, 77,5%) (Figs 5 and 6G), meaning they have been executed in residence areas with a high chance of present arbovirus pre-exposure antibodies that may (cross-)react in serological assays (Table C in S1 Appendix). Only 9,4% of the studies received the highest score (102/1082, 9,4%) (Figs 5 and 6G), which can be explained by the extensive spread of arboviruses worldwide in which almost all areas experience increasing concurrent circulation of multiple arboviruses from different antigenic groups[70–74], meaning chances of various (cross-)reacting pre-exposure antibodies are high in most regions (Tables C and D in S1 Appendix). The same scoring pattern can be observed for travel history: almost all studies were assigned with the lowest score (1067/1082, 98,6%), which in this case was largely due to not reporting or specifying this information (941/1067, 88,2%) (Figs 5 and 6H and Table C in S1 Appendix). The age of patient or vaccination groups studied varied from children only (95/1082, 8,8%) to children and adults (323/1082, 29,9%), adults only (194/1082, 17,9%) and unknown (478/1082, 44,2%), which resulted in a slight majority of studies with the lowest score (664/1082, 61,2%) (Figs 5 and 6I and Table C in S1 Appendix). Even though the potential for cross-reactivity from vaccination is well known, this information was rarely provided; hardly any of the studies reported previous arbovirus vaccination(s), which was also the reason for receiving the lowest score (930/946, 98,3%) (Figs 5 and 6J and Table C in S1 Appendix). Testing for possible prior arbovirus infections was only done in roughly a third of the studies (348/1082, 32,2%), with only 139 studies providing evidence of primary infection of the studied individuals (139/1082, 12,8%—highest score) (Figs 5 and 6K and Table C in S1 Appendix). Overall, a poor total arbovirus background score was obtained, with the majority of studies in the lowest scoring category (D) (917/1082, 84,8%) (Figs 5 and 6L).

The study size of patient groups and/or vaccinees was generally small, varying from 1 to 10 individuals or unknown (454/1082, 42%) (lowest and low score—Group D) to 11 to 100 individuals (585/1082, 54,1%) (middle score—Group C) (Figs 5, 6M, and 6N and Table C in S1 Appendix). Only a few studies used study sizes of 101 to 200 (21/1082, 1,9%) (high score—Group B) and 201 to 1000 (22/1082, 2%) (highest score—Group A) (Figs 5, 6M, and 6N and Table C in S1 Appendix).

Combining all three categories together showed no studies in the highest total reliability group (group 1), meaning that no studies had the best possible score (Figs 5, 6O, A and Table H in S1 Appendix). Group 2 (highly reliable, 12/1082, 1,1%) and 5 (least reliable, 129/1082, 11,9%) contain the minority of studies, whereas the majority of studies is in group 3 (medium reliable, 462/1082, 42,7%) or 4 (low reliable, 479/1082, 44,3%) (Figs 5, 6O, and A and Table H in S1 Appendix). This means that most of the studies provide mid-range to low quality evidence with regards to antibody cross-reactivity results. In addition, cross-reactivity reliability of studies is not improving over time (Fig 5).

### Reliability of cross-reactivity results mapped to antigenic distance

**Fig 7:**
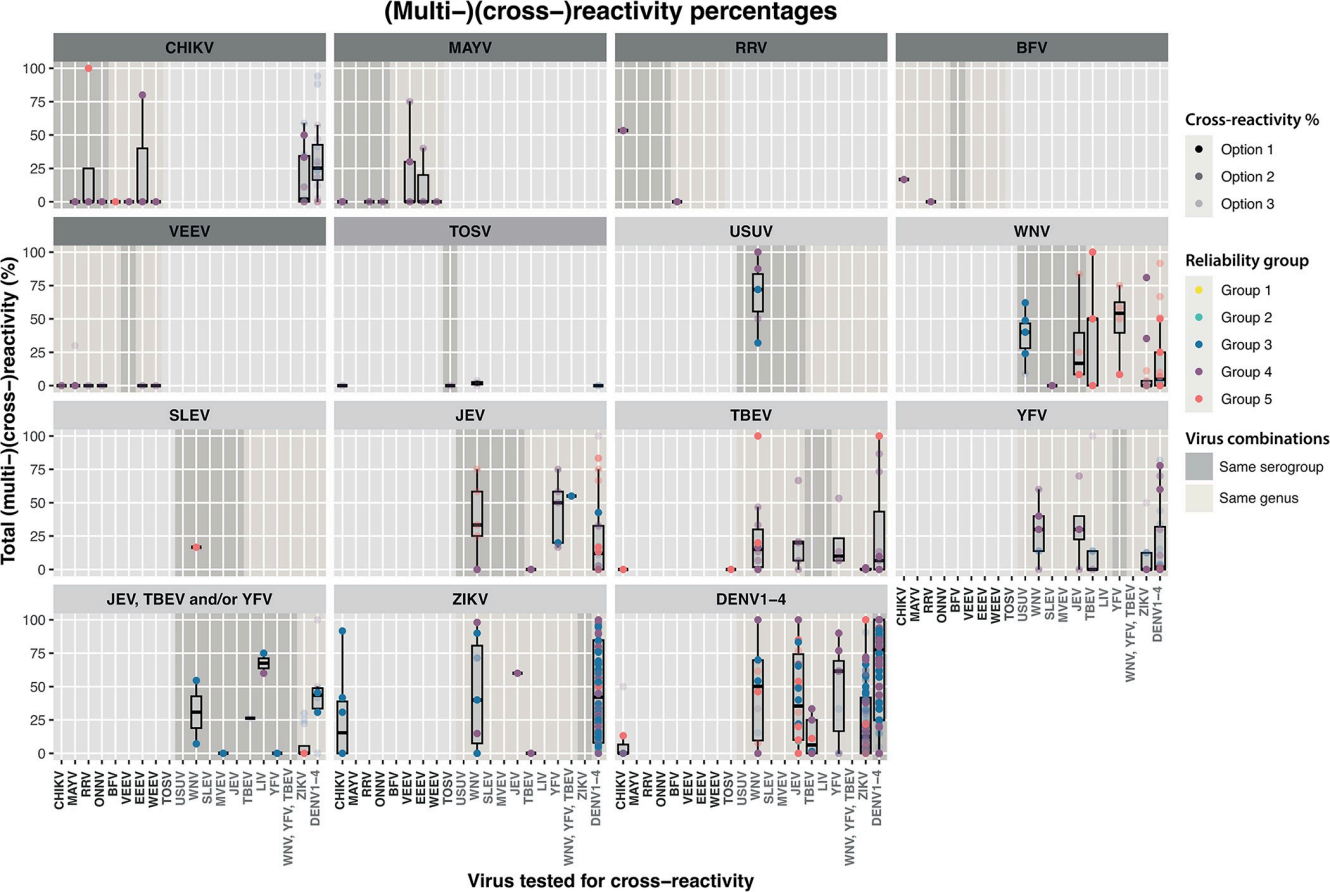
Reliability of arbovirus cross-reactivity results mapped to antigenic distance. Calculated percentage of cases exposed to different arboviruses for which (multi-)(cross-)reactive antibodies were found to viruses listed at the bottom of the panels. The heading of each panel depicts the arbovirus(es) of exposure. Coloured circles indicate the scoring from group 1 (highest) to 5 (lowest) (yellow, turquoise, dark blue, purple and pink, respectively). The transparency of the circles corresponds to the different options of calculated cross-reactivity percentages. The blue overlays depict which arboviruses tested for cross-reactivity are in the same serogroup as the arbovirus of exposure, whereas the dark grey overlays show which arboviruses are within the genus of the arbovirus of exposure. *YFV, TBEV, JEV represents all different combinations of two of these viruses or all.

The composite scores, ranging from group 1 (highest) to group 5 (lowest) were used to assess reliability of cross-reactivity results provided in the publications (Fig 7). Cross-reactivity results are presented as a calculated percentage based on the total number of confirmed patients divided into three groups (options 1–3) (see methods section and S1 Datafile). Very few papers presenting presence of antibodies to multiple arboviruses had the highest quality scores, and therefore results need to be interpreted with caution. Where tested, a medium to high percentage of serum samples tested positive for binding to other arboviruses from the same genus or serogroup, most likely reflecting cross-reactive antibodies (Fig 7). For instance, this is seen for ZIKV-DENV, DENV-DENV and to a lower extent DENV-ZIKV combinations, as well as between Tick borne encephalitis virus (TBEV), JEV, USUV, WNV and DENV1-4, in line with literature and reported protein identity [71,75–79]. However, there also are also some exceptions: a few publications on CHIKV exposed patients described low to medium levels of antibodies to ZIKV and DENV and the other way around (Fig 7). CHIKV is in a different family of viruses than ZIKV and DENV, and the studies were all in the medium or two lowest reliability groups (Fig 7). Also, most of the highest cross-reactivity percentages seen for these virus combinations are categorized as option 3 percentages, and are therefore a less accurate representation of antibody cross-reactivity (Fig 7). Therefore, it is more likely that these observations are explained by multi-reactivity caused by pre-exposure virus-specific antibodies, rather than cross-reactivity of antibodies from the recent exposure, since CHIKV, ZIKV and DENV largely co-circulate [72] (Table D in S1 Appendix). Another clear observation is that cross-reactivity between arboviruses from a different genus is rarely studied (Fig 7). In 264 cases it was not possible to calculate cross-reactivity percentages due to missing detailed information regarding the number of cross-reactive cases and/or positive confirmed patients (264/1082, 24,4%) (S1 Datafile). Similarly, assessing whether estimates differed by serological method was not possible due to the large heterogeneity of information regarding the controls, cut-offs and/or sample timing of sera used, or because information was missing or not sufficiently specified in the selected publications (e.g, type of antigen used or timing of sera sampling) (S1 Datafile).

## Discussion

Differential arbovirus diagnosis is becoming increasingly difficult due to the ongoing global arbovirus spread and, as a result, the increasingly complex antibody landscapes in exposed groups. In this systematic review, we assess the quality of information from studies aiming to assess arbovirus antibody cross-reactivity patterns, and highlight the limitations and information gaps in arbovirus serology that challenge reliable cross-reactivity results. First of all, one of the clearest knowledge gaps is that, other than ZIKV-infection and DENV-infection-induced (cross-)reactive antibody responses, antibody relations between other arboviruses are rarely studied. This underlines the need for studying cross-reactivity between these other arboviruses and broaden our knowledge beyond ZIKV and DENV to fully understand the complex antibody patterns in order to improve differential diagnosis and interpretation of serosurveys [3–7]. However, studying cross-reactivity between less well known and/or prevalent arboviruses comes with the lack of sufficiently powered clinical cohorts, lack of commercially available reliable serological tests, and the lack of standardisation and quality control panels for in-house assays.

In addition to this gap of knowledge, there is a geographic bias as well; where geographic areas such as South-, Central- or North America, Europe and South-east Asia are extensively studied, far less literature is available for patients from or travellers to Africa, West and Central Asia and Oceania, even though arboviruses are known to circulate in these areas (in the past) as well. This is in line with other literature stating arbovirus circulation in Africa, Central Asia and Oceania is likely underreported and/or underrecognized [80–82]. This may (partly) be explained by the popularity of travel destinations [83], the lack of medical facilities and/or research capabilities [84,85], limited surveillance and official reporting of arbovirus cases of regional laboratories to the WHO [84,85], and scarce availability of English reports on surveillance data in some countries of these areas [80–82].

We show that the majority of publications lack information that would allow reliable interpretation of antibody (cross-)reactivity. For instance, geographic region, travel- and vaccination and infection history of participants should be included by default in future articles studying cross-reactivity, to be able to appropriately interpret the results [70]. We show that knowledge on the background seroprevalence of different arboviruses in different parts of the world is lacking, which could be addressed by globally standardized serological studies with multi-antigen panels [70]. Such studies could serve as a global reference for researchers involved in local studies, as has been recommended for other pathogens as well [86,87]. In addition to the significant issue of missing data, we show that even in case the residence and travel areas are known, studies rarely met the highest reliability score for these parameters. The establishment of geographically dispersed longitudinal prospective or retrospective cohort studies would significantly improve our knowledge and clarify (complex) cross-reactivity results, since in this case occurrence of pre-exposures do not need to be estimated. However, this requires highly specific molecular and/or serology-based arbovirus diagnosis, clear and comprehensive registration in medical systems, and funding to allow properly powered studies beyond a single region.

Overall, most studies scored high on diagnostic specificity (group A, 736/1082, 68%), which is mainly explained by our selection of studies using vaccinees or (partially) molecular or viral-isolation methods to confirm infection of patients of at least one of the study groups that were used for testing cross-reactivity in their study. Of the studies solely or partially using serology methods to confirm virus exposures, only a few were assigned with the highest score A, and 38% of the studies were in the second highest scoring category B (137/361, 38%). Reasons for this lower score were the limited use of high-quality paired serum sample testing, lack of information regarding sampling timepoints and method specificity, as well as choosing serological diagnostic ELISA, IFA, HI or rapid diagnostic assays over more specific (confirmation) VNT assays. The method specificity issue here is, however, likely explained by the absence of available specific assays in most cases or most reliable serological tests being time consuming, mostly require large quantities of sera, are difficult to deploy in low resource settings and require highly trained personnel. Although not described in the results presented here, there were limitations in estimating and scoring this diagnostic specificity due to inconsistent or incomplete description of the methodology. For instance, many different cut-offs for serological assays were used throughout the publications, or cut-offs were not reported, the use of (negative and positive) controls was poorly defined, as were the details of sampling (single and paired sera timepoints; S1 Datafile). Although obvious, the collection of properly paired serum samples is notoriously difficult, even in costly clinical studies in high-income countries. Based on these limitations, it was not possible to include these parameters in the diagnostic specificity category. This greatly underlines the need of standardization when possible and detailed reporting of method and study characteristics.

Although there are some limitations, this reliability-scoring approach may help to pinpoint weak and strong points of studies regarding reliability of cross-reactivity signals. The observation that the total reliability score of studies is not improving over time, clearly underlines the need of more standardized and reliable study designs to be able to compare cross-reactivity study results from multiple different studies. Therefore, to overcome this, we propose minimal standards for the reporting of metadata of the studied individuals and methods used (Tables 2, 3, I, and J in S1 Appendix—blank format and recommendations). This includes detailed background information on the population tested such as the residence area or travel locations in the past years and the arbovirus exposure history when available, and preferentially studying cross-reactivity in populations that are least likely biased from possible previous arbovirus exposures. When possible, the use of specific (serological or molecular) methods with standardized control panels and cut-offs as well as standardized study designs that include paired sera sampling regarding serology and multi-antigen test panels, would highly improve the diagnostic certainty and comparability. This may eventually lead the way to improving our understanding of the complex cross-reactive antibody patterns between (less studied but relevant) arboviruses and how these responses affect disease outcome. Furthermore, these insights in cross-reactive antibody responses may help develop specific serological assays and subsequently improve differential arbovirus diagnosis, tackling the challenge of arbovirus diagnosis in a rapidly changing world regarding global arbovirus spread.

**Table 2:** A minimum standard for metadata regarding the studied individuals.

Variable	Description
**Virus of exposure**	The virus to which the individuals included in the study are exposed (infection or vaccination)
**Residence area** (Country, state, city, district)	The country and additional information about the residence of the individuals included in the study
**Travel area of travellers** (Country, state, city, district)	The area of acquired infection of travellers included in the study
**Prior travel history** (non-travellers & travellers) (Country, state, city, district)	The prior travel history of the individuals included in the study
**Prior arbovirus vaccination history**	The prior arbovirus vaccination history of individuals included in the study
**Age**	The age of the individuals (children, adults or both with the range of ages)
**Prior arbovirus infection history**	Evidence of no or prior infection history by serological or molecular methods

**Table 3:** A minimum standard for metadata regarding the methods used to confirm the virus exposure, determine the prior infection history and test for antibody cross-reactivity.

Variable	Description	Category		
		*Confirmation of arbovirus exposure*	*Assessment of arbovirus infection history*	*Testing antibody cross-reactivity*
**Methods used**	The molecular and/or serological methods used to confirm the virus exposure or determine the infection history of the individuals. The serological methods used to test antibody cross-reactivity.	x	x	x
**Cut-off used**	The cut-off used to determine whether the test result is positive or negative	x	x	x
**Type of antibody tested** *(serological methods only)*	The type of antibody tested in serological assays	x	x	x
**Antigen tested** *(serological methods only)*	The type of antigen used	x	x	x
**Case definition**	Case definition used to determine the virus of exposure	x		
**Definition of infection history status**	Interpretation of test results with regards to the infection history status		x	
**Timepoints of serum samples**	The timing of sera sampling used in the case definition, to determine the infection history and used in the cross-reactivity testing	x	x	x
**Controls used**	The negative and positive controls used	x	x	x
**Raw data**	The raw data output of the method(s) used	x	x	x
**# Total confirmed positives**	The number of total confirmed exposed individuals used to test antibody cross-reactivity			x
**# Confirmed positives that show reactivity to other antigens**	The number of confirmed exposed individuals that show reactivity to both the virus of infection and the virus tested for cross-reactivity			x

## Supporting information

S1 PRISMA Checklist PRISMA 2020 main checklist. (DOCX)

S1 Appendix Supplemental information. **A–Additional information regarding the reliability scoring system**. Information about scoring the three categories: diagnostic specificity, arbovirus background and study size, as well as the overall data quality score. **Table A–Search strategy of three different databases**. Three different databases that cover all scientific articles were used to search articles for this systematic literature search. We aimed to only select articles studying human arbovirus antibody cross-reactivity in serological assays. Reviews, meta-analyses, and case reports were excluded from the selection. **Table B–Included articles with their references**. The “Study_number” corresponds to the study numbers in the S1 Datafile. For all details about the results of scoring and the subdivided datasets, see S1 Datafile. **Table C–Overview reliability scoring system**. Variables scored in this reliability scoring system were classified in three main categories: Diagnostic specificity, arbovirus background and study size. Maximum number of points for type of test and confirmation was 40, whereas for all other variables this was 4, or 8 or 2 based on the weight of the variable. Only studies that received either 18 or 0 points in the type of test and confirmation variable, were further scored for the other serological variables of the diagnostic specificity score. The highest possible score of each variable correlates with the lowest bias by diagnostic specificity, arbovirus background and study size. This means that in this case, the diagnosis of study groups can be viewed as correctly determined and true, as well as the antibody cross-reactivity results presented by the study. **Table D–Circulation of arboviruses per area used in scoring system**. All arboviruses circulating in specific geographic areas, according to Cleton et al [70,74], additional literature [16,71–73,88–106], and CDC and WHO circulation maps, were used to calculate chances of present pre-exposure antibodies in residence and travel areas of study participants. For arboviruses that only recently circulate, the reported year of the start of circulation in particular areas was taken into account. For calculating the effect of possible pre-exposure antibodies on the antibody cross-reactivity results, DENV and ZIKV were considered as the same serogroup based on their high antigenic similarities. **Table E–Classification of residence and travel areas per geographic area**. All residence and travel areas of the literature search were classified in determined geographic regions that have a similar composition of arbovirus circulation based on Cleton et al [70,74] and CDC and WHO circulation maps. **Table F–Category groups of sum of points**. Total sum of points of each category was divided into four quartiles (Group A, B, C and D) to be able to equally weigh and compare the total scores of the different categories. **Table G–Overview combinations A, B, C and D of the three categories scoring reliability**. The table shows an overview of all the possible combinations and the found combinations (A, B, C and D) of the three main categories scoring reliability of the included studies (N = 1082). The table is ordered by showing the best possible reliable combination at the top of the table and the least possible reliable combination at the bottom of the table. **Table H–Overview of total reliability groups based on category combination score**. Total reliability groups were defined per category combination score of A, B, C and D for included studies (N = 1082). Total reliability group 1 is most reliable, whereas total reliability group 5 is the least reliable. Number of studies per total reliability group can be seen in the last column. **Table I—A minimum standard for metadata regarding the studied individuals**. **Table J—A minimum standard for metadata regarding the methods used to confirm the exposure, determine the prior infection history and test for antibody cross-reactivity. Fig A–Three-dimensional plots of category scores and total reliability groups**. Scores for all three categories (study size, diagnostic specificity and arbovirus background) in three-dimensional plots (N = 1082). Total reliability groups are depicted in colours ranging from group 1 (highest) to group 5 (lowest) (yellow, turquoise, dark blue, purple and pink, respectively). Frequency of a specific score is shown by size.
(DOCX)

S1 Datafile Extracted data of all included articles and scoring. (XLSX)
