# Supplementary material for: The increasing complexity of arbovirus serology: An in-depth systematic review on cross-reactivity
Source: PLoS Negl Trop Dis. 2023 Sep 22;17(9):e0011651. doi: 10.1371/journal.pntd.0011651 (PMC10550177; doi:10.1371/journal.pntd.0011651)
Supplement: S1 Appendix — A–Additional information regarding the reliability scoring system. Information about scoring the three categories: diagnostic specificity, arbovirus background and study size, as well as the overall data quality score. Table A–Search strategy of three different databases. Three different databases that cover all scientific articles were used to search articles for this systematic literature search. We aimed to only select articles studying human arbovirus antibody cross-reactivity in serological assays. Reviews, meta-analyses, and case reports were excluded from the selection. Table B–Included articles with their references. The “Study_number” corresponds to the study numbers in the S1 Datafile. For all details about the results of scoring and the subdivided datasets, see S1 Datafile. Table C–Overview reliability scoring system. Variables scored in this reliability scoring system were classified in three main categories: Diagnostic specificity, arbovirus background and study size. Maximum number of points for type of test and confirmation was 40, whereas for all other variables this was 4, or 8 or 2 based on the weight of the variable. Only studies that received either 18 or 0 points in the type of test and confirmation variable, were further scored for the other serological variables of the diagnostic specificity score. The highest possible score of each variable correlates with the lowest bias by diagnostic specificity, arbovirus background and study size. This means that in this case, the diagnosis of study groups can be viewed as correctly determined and true, as well as the antibody cross-reactivity results presented by the study. Table D–Circulation of arboviruses per area used in scoring system. All arboviruses circulating in specific geographic areas, according to Cleton et al [70,74], additional literature [16,71–73,88–106], and CDC and WHO circulation maps, were used to calculate chances of present pre-exposure antibodies in residence and travel areas of s [file pntd.0011651.s002.docx]

**Supplementary material**

**Supplemental Information A**

***Diagnostic specificity score***

This category in the scoring system is based on the methods used for identification of patient groups, specificity of the serological methods under evaluation, case definitions and serum sampling timepoints. These variables were chosen since they are all considered important for differential arbovirus diagnosis (1). The highest score is assigned to studies which have the highest probability that the arbovirus infections of the patients in the study are correctly characterized, and therefore, the observations on serological (cross-)reactivity are considered accurate (Fig 2 and Table C in S1 Appendix). In order to give this score, we defined variables that may influence this diagnostic specificity score (1) (Table C in S1 Appendix). These variables were each scored separately and together make up the total number of points in this category.

Studies that used only vaccinees or arbovirus-infected patients who were PCR confirmed and/or confirmed by viral-isolation were assigned with the highest possible score (40 points) (Table C in S1 Appendix), since virus isolation (by cell-culture) and PCR are proven to be highly specific in detection of arbovirus infections (WHO and CDC arbovirus case definition recommendations) (1–5). Studies using a study group that was partly vaccinees or a patients’ group confirmed by virus isolation and/or PCR, and partly serologically confirmed received 18 points (Table C in S1 Appendix). Studies that used serological methods only were assigned 0 points, as well as studies that did not specify whether PCR only, serology only or both were used which were therefore considered as serological confirmed only (Table C in S1 Appendix). Both these studies that received either 18 or 0 points, were scored to assess the reliability of the used serological methods, case definitions, method specificity and sera sampling timepoints. The rationale for scoring these variables is based on WHO or CDC recommendations (1–5). With serology, since a case is considered confirmed if a screening assay is followed by evaluation with a more-specific test (1), studies that used ELISA, IFA, HI or rapid tests as screening assay and VNT as confirmation were scored highest, followed by studies using VNT only, ELISA, IFA, HI or rapid test only and lastly based on symptoms or unknown methods(6) (Table C in S1 Appendix). This is based on the general consideration of VNT as the gold standard in arbovirus serology with regards to specificity (7–10). Studies using case definitions in which they measured and compared serology responses of viruses that possibly cross-react with each other were assigned higher scores than studies testing one possible virus of infection only, as recommended for closely related arboviruses(1,4,11) (Table C in S1 Appendix). In addition, studies using paired sera and taking kinetics into account in their final conclusions of positive or negative cases were scored higher than studies using single sera only to confirm the virus of infection (1,4,11) (Table C in S1 Appendix). By using paired sera, information about the individual immunological background can be provided to differentiate prior and recent exposures, as well as information to distinguish possible reactivity from cross-reactivity(1,4,11). In the composite score, timing of the serum sample collection was also included; studies received a higher score when they used single samples after day 10 since symptom onset when it is possible to detect the induced antibody response, or when they used paired sera before day 7 since symptom onset and after day 14 since symptom onset in order to look at antibody kinetics, than studies that did not. Additionally, serological method specificity was also scored in which studies using diagnostic assays measuring more-specific (e.g., NS1) protein binding or using additional binding-inhibition or -competition steps to increase specificity received higher points than other assays (Table C in S1 Appendix) as these are considered more specific (1,4,12–18). Of all variables scoring the serological diagnostic specificity, the type of serology method(s) was weighted highest compared to the other variables, since serological diagnostic quality by type of methods is best described in literature and often used in recommended case definitions by the WHO or CDC(1,6).

***Arbovirus background score***

The arbovirus background score is based on the ability to assess whether antibodies from previous exposure could be present in vaccinee/patient-sera, thereby biasing the cross-reactivity signal reported in the studies (Fig 2 and Table C in S1 Appendix). The highest score is assigned to studies which used vaccinee/patient-sera from which probability of bias by previous exposure antibodies is lowest, therefore most likely yielding a true cross-reactivity signal (Fig 2 and Table C in S1 Appendix). The cross-reactivity signals reported in studies which received high scores, are therefore highly likely the result of antibodies induced by the arbovirus infection of the patient or vaccination rather than previous exposure antibodies. These specific cross-reactivity signals can therefore be referred to as true cross-reactivity (Fig 2 and Table C in S1 Appendix). Conversely, lower scoring studies involve individuals with a higher chance of a combination of present previous exposure antibodies and antibodies from the recent exposure (multi(-cross)-reactivity). As recommended (1), components of this score are information regarding the geographic region of residence, travel histories, and arbovirus vaccination and infection history of individuals, as well as likelihood of bias from previous exposure antibodies by age, induced upon infection with viruses to which patients or vaccinees could have been exposed.

To assess probability of bias by antibodies induced by previous exposure to arboviruses in residence or travel areas, the circulating arboviruses per area were listed based on our previous articles (19,20), as well as additional literature (21–43) and WHO and CDC circulation maps (Table D in S1 Appendix). Subsequently, the residence area and travel history of patients or vaccinees were matched to this list of circulating viruses per geographic area, based on Table E in S1 Appendix. A virus circulating in the vaccinees’ or patients’ residence or travel area which is the same virus as tested for cross-reactivity in serological assays, resulted in a low reliability score since these individuals may be previously exposed to this virus and in this case, it is highly likely that antibodies induced by these previous exposure(s) bias the cross-reactivity signal ((Fig 2 and Table C in S1 Appendix). Therefore, the signal is referred to as multi-reactivity rather than cross-reactivity, since the measured serological signals are likely the result of previously induced virus-specific antibodies with or without cross-reactive antibodies from recent exposure to the virus of infection or vaccination (Table C in S1 Appendix). Generally, it is assumed that the more antigenic homology arboviruses share, the more antibody cross-reactivity can be seen. Thus, bias by previously induced antibodies of virus(es) circulating in the residence or travel area that are in the same serogroup as the virus tested for cross-reactivity is highly likely, since these arboviruses share antigenic properties and are closely related. This means that here, you expect cross-reactive antibodies from previous exposure and possibly cross-reactive antibodies from the recent infection or vaccination, referred to as multi-cross-reactivity (Fig 2 and Table C in S1 Appendix). Lastly, a circulating virus which is in a different serogroup as the virus tested for cross-reactivity suggests that probability of previous exposure antibodies biasing the cross-reactivity signal is low. Therefore, the signal can be referred to as true cross-reactivity (Fig 2 and Table C in S1 Appendix).

The same principle as described for the residence area and travel history of patients or vaccinees, applies to arbovirus vaccination history; antigenic properties of virus(es) of previous exposure by vaccination are compared to the virus tested for antibody cross-reactivity to determine risk of bias (Fig 2 and Table C in S1 Appendix). If studies reported that studied patients or vaccinees did not have any travel history or did not receive any previous (relevant) arbovirus vaccinations, these studies were assigned the highest possible score in the travel and arbovirus vaccination history variables since these individuals have lower probability of biasing the cross-reactivity signal by previous exposure antibodies from vaccination or travel (Table C in S1 Appendix). Age is also taken into account by assigning lower scores to studies using adults than studies using children, since probability of (multiple) previous arbovirus exposures increase by lifetime (1,44) (Table C in S1 Appendix). In addition, studies were scored on whether they provided evidence of the arbovirus infection status of the studied individuals. Studies that tested for possible prior exposure antibodies in acute sera, or followed the individuals in time and kept track of the molecular and/or serological confirmation of previous arbovirus infections, and reported that all individuals were arbovirus naive (primary infection), received the highest score. Studies that did not look into the infection history or reported evidence of prior arbovirus exposures, received the lowest score since the (possible) pre-exposure antibodies may bias the cross-reactivity signals. Overall, the residence area of patients and/or vaccinees is weighted highest among the other variables, since individuals can be infected with (multiple) circulating arboviruses life-long in this area, and therefore chances of bias are highest for this variable.

***Study size score***

Overall cross-reactivity reliability was scored by study size; a study size of 1 to 10 or unknown number of patients and/or vaccinees, was considered as low reliable and least reliable respectively, whereas the highest reliability score was given to studies using larger study groups of 201 to 1000 individuals (Table C in S1 Appendix).

***Overall data quality score***

To obtain the total score per category per study, we used the sum of points of each variable. Subsequently, to equally weigh each category, we ranked these total category scores by dividing each category into four quartiles (A, B, C and D), of which A is the highest and D the lowest scoring group (Table F in S1 Appendix). For the final reliability score, all different combinations of A, B, C and D per category were ranked from best possible combinations to worst possible combinations (Table G in S1 Appendix) and subsequently divided into a final ranking of 5 groups (Table H in S1 Appendix). Final reliability group 1 includes studies that generated the most reliable antigenic cross-reactivity results whereas group 5 is scored as presenting the least reliable results in regards to cross-reactivity of antibodies (Table H in S1 Appendix).

**Supplemental tables**

| **embase.com** | ('Arbovirus'/de OR 'Flaviviridae infection'/de OR 'Flaviviridae'/de OR 'Flavivirus'/exp OR 'Flavivirus infection'/exp OR 'Bunyaviridae'/exp OR 'bunyavirus infection'/exp OR 'Nairovirus'/exp OR 'Nairovirus infection'/exp OR 'Orthobunyavirus'/exp OR 'Orthobunyavirus infection'/exp OR 'Phlebovirus'/exp OR 'Phlebovirus infection'/exp OR 'Reoviridae'/exp OR 'reovirus infection'/exp OR 'Sedoreovirinae'/exp OR 'Spinareovirinae'/exp OR 'Orbivirus'/exp OR 'Coltivirus'/exp OR 'Togaviridae'/exp OR 'togavirus infection'/de OR 'Alphavirus'/exp OR 'Alphavirus infection'/exp OR 'tick borne encephalitis'/exp OR 'Rift Valley fever virus'/de OR 'West Nile virus'/exp OR (Arbovir* OR Arbo-vir* OR (Arthropod* NEAR/3 virus*) OR Bunyavir* OR Nairovir* OR orthonairovir* OR Orthobunyavir* OR Phlebovir* OR Flavivir* OR Flavivir* OR Reovir* OR Sedoreovir* OR Orbivir* OR Seadornavir* OR Spinareovir* OR Coltivir* OR Togavir* OR Alphavir* OR ((tick OR mosquito OR vector) NEAR/3 (borne) NEAR/3 (virus*)) OR dengue OR denv OR zika OR zikv OR ((Rift-Valley OR yellow) NEAR/3 fever) OR hantavir* OR Chikungunya OR west-nile OR mayaro OR (Japanese* NEAR/3 encephalit*) OR Crimean-Congo):ab,ti) AND ('cross reaction'/de OR 'cross reacting antibody'/de OR 'cross reacting antigen'/de OR (((cross OR broad OR non-specific OR nonspecific) NEXT/1 reacti*) OR (serolog* NEAR/3 assay*)):ab,ti) NOT ('review'/exp OR 'meta analysis'/exp OR 'case report'/de OR (review OR 'meta analysis' OR 'case report*'):ti) NOT (antibod* NEAR/3 depend* NEAR/3 enhance*) NOT ([Conference Abstract]/lim OR [letter]/lim OR [note]/lim OR [editorial]/lim) AND [English]/lim AND ('observational study'/exp OR 'cohort analysis'/exp OR 'longitudinal study'/exp OR 'retrospective study'/exp OR 'prospective study'/exp OR 'health survey'/de OR 'health care survey'/de OR 'epidemiological data'/de OR 'case control study'/de OR 'population research'/de OR 'major clinical study'/de OR 'multicenter study'/de OR 'follow up'/de OR 'open study'/de OR (((observation* OR epidemiolog* OR communit*) NEAR/6 (stud* OR data OR research)) OR cohort* OR longitudinal* OR retrospectiv* OR prospectiv* OR population* OR (national* NEAR/3 (stud* OR survey)) OR (health* NEAR/3 survey*) OR ((case OR cases OR match*) NEAR/3 control*) OR multicenter* OR multi-center* OR follow-up* OR followup*):ab,ti) |
| --- | --- |
| **Medline Ovid** | (Arboviruses/ OR exp Arbovirus Infections/ OR Flaviviridae/ OR exp Flavivirus/ OR Flaviviridae Infections/ OR exp Flavivirus Infections/ OR exp Bunyaviridae/ OR exp Bunyaviridae Infections/ OR exp Reoviridae/ OR exp Reoviridae Infections/ OR exp Togaviridae/ OR exp Togaviridae Infections/ OR (Arbovir* OR Arbo-vir* OR (Arthropod* ADJ3 virus*) OR Bunyavir* OR Nairovir* OR orthonairovir* OR Orthobunyavir* OR Phlebovir* OR Flavivir* OR Flavivir* OR Reovir* OR Sedoreovir* OR Orbivir* OR Seadornavir* OR Spinareovir* OR Coltivir* OR Togavir* OR Alphavir* OR ((tick OR mosquito OR vector) ADJ3 (borne) ADJ3 (virus*)) OR dengue OR denv OR zika OR zikv OR ((Rift-Valley OR yellow) ADJ3 fever) OR hantavir* OR Chikungunya OR west-nile OR mayaro OR (Japanese* ADJ3 encephalit*) OR Crimean-Congo).ab,ti.) AND (cross reaction/ OR cross reacting antibody/ OR cross reacting antigen/ OR (((cross OR broad OR non-specific OR nonspecific) ADJ reacti*) OR (serolog* ADJ3 assay*)).ab,ti.) NOT (review/ OR meta analysis/ OR case report/ OR (review OR meta analysis OR case report*).ti.) NOT (antibod* ADJ3 depend* ADJ3 enhance*) NOT (letter* OR news OR comment* OR editorial* OR congres* OR abstract* OR book* OR chapter* OR dissertation abstract*).pt. AND english.la. AND (Observational Study/ OR exp Cohort Studies/ OR Health Care Surveys/ OR Health Surveys/ OR Case-Control Studies/ OR Multicenter Study/ OR follow up/ OR (((observation* OR epidemiolog* OR communit*) ADJ6 (stud* OR data OR research)) OR cohort* OR longitudinal* OR retrospectiv* OR prospectiv* OR population* OR (national* ADJ3 (stud* OR survey)) OR (health* ADJ3 survey*) OR ((case OR cases OR match*) ADJ3 control*) OR multicenter* OR multi-center* OR follow-up* OR followup*).ab,ti.) |
| **Web of science** | TS=(((Arbovir* OR Arbo-vir* OR (Arthropod* NEAR/2 virus*) OR Bunyavir* OR Nairovir* OR orthonairovir* OR Orthobunyavir* OR Phlebovir* OR Flavivir* OR Flavivir* OR Reovir* OR Sedoreovir* OR Orbivir* OR Seadornavir* OR Spinareovir* OR Coltivir* OR Togavir* OR Alphavir* OR ((tick OR mosquito OR vector) NEAR/2 (borne) NEAR/2 (virus*)) OR dengue OR denv OR zika OR zikv OR ((Rift-Valley OR yellow) NEAR/2 fever) OR hantavir* OR Chikungunya OR west-nile OR mayaro OR (Japanese* NEAR/2 encephalit*) OR Crimean-Congo)) AND ((((cross OR broad OR non-specific OR nonspecific) NEAR/1 reacti*) OR (serolog* NEAR/2 assay*))) NOT (antibod* NEAR/2 depend* NEAR/2 enhance*) AND ((((observation* OR epidemiolog* OR communit*) NEAR/5 (stud* OR data OR research)) OR cohort* OR longitudinal* OR retrospectiv* OR prospectiv* OR population* OR (national* NEAR/2 (stud* OR survey)) OR (health* NEAR/2 survey*) OR ((case OR cases OR match*) NEAR/2 control*) OR multicenter* OR multi-center* OR follow-up* OR followup*))) NOT TI=((review OR "metaanalysis" OR "case report*")) AND DT=(article) AND LA=(english) |

**Table A – Search strategy of three different databases**Three different databases that cover all scientific articles were used to search articles for this systematic literature search. We aimed to only select articles studying human arbovirus antibody cross-reactivity in serological assays. Reviews, meta-analyses, and case reports were excluded from the selection.

| Study_number (corresponds to study number in S1 Datafile) | Reference | Number of subdivided datasets used in scoring system (see S1 Datafile for all details, including study scores) |
| --- | --- | --- |
| 1 | (45) | 3 |
| 2 | (46) | 4 |
| 3 | (47) | 1 |
| 4 | (48) | 6 |
| 5 | (49) | 3 |
| 6 | (50) | 21 |
| 7 | (51) | 18 |
| 8 | (18) | 15 |
| 9 | (52) | 9 |
| 10 | (53) | 4 |
| 11 | (54) | 10 |
| 12 | (55) | 17 |
| 13 | (56) | 16 |
| 14 | (57) | 6 |
| 15 | (58) | 7 |
| 16 | (12) | 1 |
| 17 | (59) | 2 |
| 18 | (60) | 2 |
| 19 | (61) | 12 |
| 20 | (62) | 16 |
| 21 | (63) | 2 |
| 22 | (64) | 2 |
| 23 | (65) | 2 |
| 24 | (66) | 6 |
| 25 | (67) | 3 |
| 26 | (68) | 12 |
| 27 | (69) | 9 |
| 28 | (70) | 8 |
| 29 | (71) | 9 |
| 30 | (72) | 9 |
| 31 | (73) | 6 |
| 32 | (74) | 9 |
| 33 | (75) | 2 |
| 34 | (31) | 2 |
| 35 | (76) | 18 |
| 36 | (77) | 2 |
| 37 | (78) | 24 |
| 38 | (79) | 2 |
| 39 | (80) | 64 |
| 40 | (81) | 10 |
| 41 | (82) | 6 |
| 42 | (83) | 6 |
| 43 | (84) | 10 |
| 44 | (85) | 8 |
| 45 | (86) | 2 |
| 46 | (87) | 1 |
| 47 | (88) | 1 |
| 48 | (89) | 8 |
| 49 | (90) | 4 |
| 50 | (91) | 72 |
| 51 | (92) | 6 |
| 52 | (93) | 28 |
| 53 | (94) | 5 |
| 54 | (95) | 2 |
| 55 | (96) | 55 |
| 56 | (97) | 3 |
| 57 | (98) | 8 |
| 58 | (13) | 4 |
| 59 | (99) | 2 |
| 60 | (100) | 3 |
| 61 | (101) | 1 |
| 62 | (102) | 2 |
| 63 | (103) | 51 |
| 64 | (104) | 6 |
| 65 | (105) | 10 |
| 66 | (106) | 30 |
| 67 | (107) | 63 |
| 68 | (108) | 12 |
| 69 | (109) | 11 |
| 70 | (110) | 11 |
| 71 | (111) | 20 |
| 72 | (112) | 1 |
| 73 | (113) | 8 |
| 74 | (114) | 10 |
| 75 | (115) | 44 |
| 76 | (116) | 10 |
| 77 | (117) | 5 |
| 78 | (118) | 2 |
| 79 | (119) | 2 |
| 80 | (120) | 6 |
| 81 | (121) | 6 |
| 82 | (122) | 28 |
| 83 | (123) | 30 |
| 84 | (124) | 7 |
| 85 | (125) | 2 |
| 86 | (126) | 6 |
| 87 | (127) | 4 |
| 88 | (128) | 4 |
| 89 | (129) | 6 |
| 90 | (130) | 6 |
| 91 | (131) | 4 |
| 92 | (132) | 16 |
| 93 | (133) | 4 |
| 94 | (134) | 5 |
| 95 | (135) | 35 |
| 96 | (136) | 8 |
| 97 | (137) | 4 |
| 98 | (138) | 2 |
| 99 | (139) | 6 |
| 100 | (140) | 11 |
| 101 | (141) | 7 |
| 102 | (142) | 8 |

**Table B – Included articles with their references**The “Study number” corresponds to the study numbers in S1 Datafile. For all details about the results of scoring and the subdivided datasets, see S1 Datafile.

|  |  | **Reliable Not reliable** | | | | |  | |
| --- | --- | --- | --- | --- | --- | --- | --- | --- |
|  |  |  |  |  |  |  |  | |
|  |  | **Highest score** | **High score** | **Middle score** | **Low score** | **Lowest score** |  |  |
|  |  |  |  |  |  |  |  |  |
|  | **Weight** | **Correct virus (infection) confirmation** |  |  |  | **Incorrect virus (infection) confirmation** | **Maximum score** |  |
| **Diagnostic specificity score** |  |  |  |  |  |  | **40** |  |
| Points |  | **40** |  | **18** |  | **0** |  |  |
| Type of test and confirmation | 1 | Vaccinees or all patients of study are confirmed by PCR and/or virus-isolation (by cell-culture) | NA | Partly vaccinees or part of patient study group is confirmed by PCR and/or virus-isolation (by cell-culture). Rest of patients are serologically confirmed | NA | All patients of study are serologically confirmed, or not specified whether patients were confirmed by PCR only, serology only or both | 40 |  |
| **Studies with 20 or 0 points in “Type of (infection) confirmation” variable are scored by serological diagnostic specificity variables below** |  |  |  |  |  |  | 18 |  |
| Points |  | **4** | **3** | **2** | **1** | **0** |  |  |
| Serological case definition used  *(if there was a combination, the highest category was chosen)* | 1 | Paired sera - Seroconversion or 4-fold increase IgM/IgG & lower (increasing) titers of antibodies directed towards virus of which antibodies likely cross-react | Paired sera -Seroconversion or 4-fold increase IgM/IgG | Single sera (or unknown) - IgM/IgG above cut-off and negative result for, or 4-fold difference with, virus of which antibodies likely cross-react | Single sera (or unknown) - IgM/IgG above cut-off and higher result than virus of which antibodies likely cross-react | Single sera (or unknown) -IgM/IgG above cut-off or unknown | 4 |  |
| Sera sampling timepoints | 0,5 | All samples: Single serum after 10 dpso or paired sera before 7 & after 14 dpso |  | Part of samples: Single serum after 10 dpso or paired sera before 7 & after 14 dpso |  | Other timepoints or unknown (not reported) | 2 |  |
| Type of serology method(s)  *(if there was a combination, the highest category was chosen)* | 2 | Screening assay ELISA/IFA/HI/rapid test + VNT confirmation | NA | VNT only | ELISA/IFA/HI/rapid test only | Unknown or based on symptoms | 8 |  |
| Serological method specificity *(if there was a combination, the highest category was chosen)* | 1 | *More-specific (mutated) antigens:* e.g., in case of flaviviruses NS1 (Whole NS1, mutated more-specific NS1 or NS1 in competition, inhibition or BOB ELISA) | *Conserved antigens that are mutated or used with additional steps to increase specificity: e.g.,* in case of flaviviruses or alphaviruses E (or unknown) in competition or inhibition ELISA, or mutated more-specific E proteins (e.g., EDIII, Equad) | Whole E protein | Whole virus (or lysate of whole virus/mixed proteins) | Unknown (not reported) | 4 |  |
|  |  |  |  |  |  |  |  |  |
|  |  |  |  |  |  |  |  |  |
|  |  | **Cross-reactivity** |  |  |  | **Multi-reactivity** |  |  |
| **Arbovirus background score** |  |  |  |  |  |  | **24** |  |
| Points |  | **4** | **3** | **2** | **1** | **0** |  |  |
| Residence area | 2 | Virus tested for cross-reactivity and (all) virus(es) circulating in residence area are in a different antigenic serogroup (during and before time of sample collection) | NA | Virus tested for cross-reactivity and (1 or more) virus(es) circulating in residence area are in the same antigenic serogroup (during and before time of sample collection) | NA | Virus tested for cross-reactivity is the same as (1 of) the virus(es) circulating in residence area (during and before time of sample collection) or residence area is unknown/not reported | 8 |  |
| Travel history | 1 | No previous travel of individuals | NA | Previous/recent travel to area is endemic for virus(es) that are in a different antigenic serogroup as the virus tested for cross-reactivity | Previous/recent travel to area is endemic for virus(es) that are in the same antigenic serogroup as the virus tested for cross-reactivity | Previous/recent travel to area is endemic for the virus tested for cross-reactivity or travel history is unknown/not reported | 4 |  |
| Age | 1 | Children | NA | Children and adults or children and unknown | NA | Adults or unknown/not reported | 4 |  |
| Arbovirus vaccination history | 1 | No previous (relevant) arbovirus vaccinations *(relevant is considered as a vaccination with a virus in the same genus as the virus tested for cross-reactivity)* | NA No previous (relevant) vaccinations with some arboviruses  *(relevant is considered as a vaccination with a virus in the same genus as the virus tested for cross-reactivity)* | Previous vaccination with virus(es) that are in a different antigenic serogroup as the virus tested for cross-reactivity | Previous vaccination with virus(es) that are in the same antigenic serogroup as the virus tested for cross-reactivity | Previous vaccination with the same virus as tested for cross-reactivity or unknown/ vaccination history not reported | 4 |  |
| Arbovirus infection history | 1 | (Serological) evidence of no prior arbovirus exposure (arbovirus-naive/primary infection) |  | (Serological) evidence of no prior arbovirus infection (arbovirus-naive/primary infection) in part of individuals |  | (Serological) evidence of prior arbovirus infection (arbovirus-experienced/secondary infection) or not reported/unknown | 4 |  |
|  |  |  |  |  |  |  |  |  |
|  |  |  |  |  |  |  |  |  |
|  |  | **Overall reliable cross-reactivity association** |  |  |  | **Overall non-reliable cross-reactivity association** |  |  |
| **Study size score** |  |  |  |  |  |  | **4** |  |
| Points |  | **4** | **3** | **2** | **1** | **0** |  |  |
| Study size | 1 | >200 | 101 - 200 | 11 - 100 | 1 - 10 | Unknown | 4 |  |

**Table C – Overview reliability scoring system**

Variables scored in this reliability scoring system were classified in three main categories: Diagnostic specificity, arbovirus background and study size. Maximum number of points for type of test and confirmation was 40, whereas for all other variables this was 4, or 8 or 2 based on the weight of the variable. Only studies that received either 18 or 0 points in the type of test and confirmation variable, were further scored for the other serological variables of the diagnostic specificity score. The highest possible score of each variable correlates with the lowest bias by diagnostic specificity, arbovirus background and study size. This means that in this case, the diagnosis of study groups can be viewed as correctly determined and true, as well as the antibody cross-reactivity results presented by the study.

| **Geographic region** | **Circulating arbovirus** | **Full name** | **Genus of circulating arbovirus** | **Serogroup of circulating arbovirus** | **Start year of circulation** |
| --- | --- | --- | --- | --- | --- |
| **Northern America** | WNV | WNV (West Nile virus) | Flavivirus | Japanese encephalitis |  |
|  | SLEV | SLEV (Saint Louis encephalitis virus) | Flavivirus | Japanese encephalitis |  |
|  | DENV | DENV (Dengue virus) | Flavivirus | Dengue virus |  |
|  | POWV | POWV (Powassan virus) | Flavivirus | Mammalian tick-borne flavivirus group |  |
|  | LACV | LACV (La Crosse virus) | Orthobunyavirus | California encephalitis |  |
|  | WEEV | WEEV (Western equine encephalitis virus) | Alphavirus | Western equine encephalitis |  |
|  | EEEV | EEEV (Eastern equine encephalitis virus) | Alphavirus | Eastern equine encephalitis |  |
|  | CHIKV | CHIKV (Chikungunya virus) | Alphavirus | Semliki forest | 2014 |
|  | CEV | CEV (California encephalitis virus) | Orthobunyavirus | California encephalitis |  |
|  | CTFV | CTFV (Colorado tick fever virus) | Coltivirus | Colorado tick fever |  |
| **Mexico, Central America and Caribbean** | ZIKV | ZIKV (Zika virus) | Flavivirus | Spondweni virus group | 2015 |
|  | WNV | WNV (West Nile virus) | Flavivirus | Japanese encephalitis |  |
|  | SLEV | SLEV (Saint Louis encephalitis virus) | Flavivirus | Japanese encephalitis |  |
|  | ILHV | ILHV (Ilheus virus) | Flavivirus | Japanese encephalitis |  |
|  | DENV | DENV (Dengue virus) | Flavivirus | Dengue virus |  |
|  | OROV | OROV (Oropouche virus) | Orthobunyavirus | Simbu |  |
|  | GROV | GROV (Guaroa virus) | Orthobunyavirus | California encephalitis |  |
|  | MAYV | MAYV (Mayaro virus) | Alphavirus | Semliki forest |  |
|  | CHIKV | CHIKV (Chikungunya virus) | Alphavirus | Semliki forest | 2013 |
|  | WEEV | WEEV (Western equine encephalitis virus) | Alphavirus | Western equine encephalitis |  |
|  | EEEV | EEEV (Eastern equine encephalitis virus) | Alphavirus | Eastern equine encephalitis |  |
|  | VEEV | VEEV (Venezuelan equine encephalitis virus) | Alphavirus | Venezuelan equine encephalitis virus |  |
| **South America** | ZIKV | ZIKV (Zika virus) | Flavivirus | Spondweni virus group | 2015 |
|  | WNV | WNV (West Nile virus) | Flavivirus | Japanese encephalitis |  |
|  | SLEV | SLEV (Saint Louis encephalitis virus) | Flavivirus | Japanese encephalitis |  |
|  | ILHV | ILHV (Ilheus virus) | Flavivirus | Japanese encephalitis |  |
|  | DENV | DENV (Dengue virus) | Flavivirus | Dengue virus |  |
|  | YFV | YFV (Yellow fever virus) | Flavivirus | Yellow fever |  |
|  | OROV | OROV (Oropouche virus) | Orthobunyavirus | Simbu |  |
|  | GROV | GROV (Guaroa virus) | Orthobunyavirus | California encephalitis |  |
|  | MAYV | MAYV (Mayaro virus) | Alphavirus | Semliki forest |  |
|  | CHIKV | CHIKV (Chikungunya virus) | Alphavirus | Semliki forest | 2014 |
|  | WEEV | WEEV (Western equine encephalitis virus) | Alphavirus | Western equine encephalitis |  |
|  | EEEV | EEEV (Eastern equine encephalitis virus) | Alphavirus | Eastern equine encephalitis |  |
|  | VEEV | VEEV (Venezuelan equine encephalitis virus) | Alphavirus | Venezuelan equine encephalitis virus |  |
| **North Africa** | WNV | WNV (West Nile virus) | Flavivirus | Japanese encephalitis |  |
|  | USUV | USUV (Usutu virus) | Flavivirus | Japanese encephalitis | 2012 |
|  | DENV | DENV (Dengue virus) | Flavivirus | Dengue virus |  |
|  | YFV | YFV (Yellow fever virus) | Flavivirus | Yellow fever |  |
|  | CHIKV | CHIKV (Chikungunya virus) | Alphavirus | Semliki forest |  |
|  | SFV | SFV (Semliki Forest virus) | Alphavirus | Semliki forest |  |
|  | SINV | SINV (Sindbis virus) | Alphavirus | Western equine encephalitis (sindbis-like) |  |
|  | RVFV | RVFV (Rift valley fever virus) | Phlebovirus | Phlebovirus fever |  |
|  | TOSV | TOSV (Toscana virus) | Phlebovirus | Phlebovirus fever |  |
|  | TAHV | TAHV (Tahyna virus) | Orthobunyavirus | California encephalitis |  |
|  | BUNV | BUNV (Bunyamwera virus) | Orthobunyavirus | Bunyamwera |  |
|  | CCHFV | CCHFV (Crimean Congo Haemorrhagic fever virus) | Nairovirus | Crimean-Congo hemorrhagic fever |  |
| **Sub-Saharan Africa** | ZIKV | ZIKV (Zika virus) | Flavivirus | Spondweni virus group |  |
|  | WNV | WNV (West Nile virus) | Flavivirus | Japanese encephalitis |  |
|  | USUV | USUV (Usutu virus) | Flavivirus | Japanese encephalitis |  |
|  | DENV | DENV (Dengue virus) | Flavivirus | Dengue virus |  |
|  | YFV | YFV (Yellow fever virus) | Flavivirus | Yellow fever |  |
|  | CHIKV | CHIKV (Chikungunya virus) | Alphavirus | Semliki forest |  |
|  | SINV | SINV (Sindbis virus) | Alphavirus | Western equine encephalitis (sindbis-like) |  |
|  | ONNV | ONNV (O'nyong'nyong virus) | Alphavirus | Semliki forest |  |
|  | TAHV | TAHV (Tahyna virus) | Orthobunyavirus | California encephalitis |  |
|  | BUNV | BUNV (Bunyamwera virus) | Orthobunyavirus | Bunyamwera |  |
|  | ILEV | ILEV (Ilesha virus) | Orthobunyavirus | Bunyamwera |  |
|  | NRIV | NRIV (Ngari virus) | Orthobunyavirus | Bunyamwera |  |
|  | BWAV | BWAV (Bwamba virus) | Orthobunyavirus | Bwamba |  |
|  | TATV | TATV (Tataguine virus) | Orthobunyavirus | Tataguine |  |
|  | RVFV | RVFV (Rift valley fever virus) | Phlebovirus | Phlebovirus fever |  |
|  | CCHFV | CCHFV (Crimean Congo Haemorrhagic fever virus) | Nairovirus | Crimean-Congo hemorrhagic fever |  |
| **Western and Central Asia** | WNV | WNV (West Nile virus) | Flavivirus | Japanese encephalitis |  |
|  | DENV | DENV (Dengue virus) | Flavivirus | Dengue virus |  |
|  | AHFV | AHFV (Alkhurma hemorrhagic fever virus) | Flavivirus | Mammalian tick-borne flavivirus group |  |
|  | TBEV | TBEV (Tick borne encephalitis virus) | Flavivirus | Mammalian tick-borne flavivirus group |  |
|  | SFV | SFV (Semliki Forest virus) | Alphavirus | Semliki forest |  |
|  | SINV | SINV (Sindbis virus) | Alphavirus | Western equine encephalitis (sindbis-like) |  |
|  | RVFV | RVFV (Rift valley fever virus) | Phlebovirus | Phlebovirus fever |  |
|  | TAHV | TAHV (Tahyna virus) | Orthobunyavirus | California encephalitis |  |
|  | BANV | BANV (Banna virus) | Seadornavirus | Bunna |  |
|  | CCHFV | CCHFV (Crimean Congo Haemorrhagic fever virus) | Nairovirus | Crimean-Congo hemorrhagic fever |  |
| **South-East, South and East Asia** | ZIKV | ZIKV (Zika virus) | Flavivirus | Spondweni virus group |  |
|  | WNV | WNV (West Nile virus) | Flavivirus | Japanese encephalitis |  |
|  | JEV | JEV (Japanese encephalitis virus) | Flavivirus | Japanese encephalitis |  |
|  | DENV | DENV (Dengue virus) | Flavivirus | Dengue virus |  |
|  | KFDV | KFDV (Kyasanur Forest disease virus) | Flavivirus | Mammalian tick-borne flavivirus group |  |
|  | TBEV | TBEV (Tick borne encephalitis virus) | Flavivirus | Mammalian tick-borne flavivirus group |  |
|  | AHFV | AHFV (Alkhurma hemorrhagic fever virus) | Flavivirus | Mammalian tick-borne flavivirus group |  |
|  | TAHV | TAHV (Tahyna virus) | Orthobunyavirus | Tataguine |  |
|  | BANV | BANV (Banna virus) | Seadornavirus | Bunna |  |
|  | SFTSV | SFTSV (Severe Fever with Thrombocytopenia Syndrome Virus) | Phlebovirus | Bhanja |  |
|  | CHIKV | CHIKV (Chikungunya virus) | Alphavirus | Semliki forest |  |
|  | SFV | SFV (Semliki Forest virus) | Alphavirus | Semliki forest |  |
| **Oceania** | ZIKV | ZIKV (Zika virus) | Flavivirus | Spondweni virus group | 2007 |
|  | JEV | JEV (Japanese encephalitis virus) | Flavivirus | Japanese encephalitis |  |
|  | WNV | WNV (West nile virus) | Flavivirus | Japanese encephalitis |  |
|  | DENV | DENV (Dengue virus) | Flavivirus | Dengue virus |  |
|  |  |  |  |  |  |
|  | MVEV | MVEV (Murray Valley encephalitis virus) | Flavivirus | Japanese encephalitis |  |
|  | KUNV | KUNV (Kunjin virus) (Subtype of WNV) | Flavivirus | Japanese encephalitis |  |
|  | BFV | BFV (Barmah Forest virus) | Alphavirus | Barmah Forest |  |
|  | SINV | SINV (Sindbis virus) | Alphavirus | Western equine encephalitis (sindbis-like) |  |
|  | CHIKV | CHIKV (Chikungunya virus) | Alphavirus | Semliki forest | 2011 |
|  | RRV | RRV (Ross River virus) | Alphavirus | Semliki forest |  |
| **Northern Europe** | TBEV | TBEV (Tick borne encephalitis virus) | Flavivirus | Mammalian tick-borne flavivirus group |  |
|  | SINV | SINV (Sindbis virus) | Alphavirus | Western equine encephalitis (sindbis-like) |  |
| **Southern Europe** | WNV | WNV (West Nile virus) | Flavivirus | Japanese encephalitis | 2010 |
|  | USUV | USUV (Usutu virus) | Flavivirus | Japanese encephalitis | 2009 |
|  | DENV | DENV (Dengue virus) | Flavivirus | Dengue virus | 2018 |
|  | SFV | SFV (Semliki Forest virus) | Alphavirus | Semliki forest |  |
|  | CHIKV | CHIKV (Chikungunya virus) | Alphavirus | Semliki forest | 2007 |
|  | TOSV | TOSC (Toscana virus) | Phlebovirus | Phlebovirus fever |  |
|  | CCHFV | CCHFV (Crimean Congo Haemorrhagic fever virus) | Nairovirus | Crimean-Congo hemorrhagic fever |  |
| **Central Europe** | TBEV | TBEV (Tick borne encephalitis virus) | Flavivirus | Mammalian tick-borne flavivirus group |  |
|  | WNV | WNV (West Nile virus) | Flavivirus | Japanese encephalitis | 2019 |
|  | USUV | USUV (Usutu virus) | Flavivirus | Japanese encephalitis | 2012 |
|  | TAHV | TAHV (Tahyna virus) | Orthobunyavirus | California encephalitis |  |
| **Western Europe** | DENV | DENV (Dengue virus) | Flavivirus | Dengue virus | 2010 |
|  | TBEV | TBEV (Tick borne encephalitis virus) | Flavivirus | Mammalian tick-borne flavivirus group |  |
|  | LIV | LIV (Louping ill virus) | Flavivirus | Mammalian tick-borne flavivirus group |  |
|  | WNV | WNV (West Nile virus) | Flavivirus | Japanese encephalitis | 2020 |
|  | USUV | USUV (Usutu virus) | Flavivirus | Japanese encephalitis | 2016 |
|  | SFV | SFV (Semliki Forest virus) | Alphavirus | Semliki forest |  |
|  | CHIKV | CHIKV (Chikungunya virus) | Alphavirus | Semliki forest | 2010 |
|  | TOSV | TOSC (Toscana virus) | Phlebovirus | Phlebovirus fever |  |
| **Eastern Europe (incl. Russia)** | WNV | WNV (West Nile virus) | Flavivirus | Japanese encephalitis |  |
|  | USUV | USUV (Usutu virus) | Flavivirus | Japanese encephalitis | 2015 |
|  | TBEV | TBEV (Tick borne encephalitis virus) | Flavivirus | Mammalian tick-borne flavivirus group |  |
|  | OHFV | OHFV (Omsk hemorrhagic fever virus) | Flavivirus | Mammalian tick-borne flavivirus group |  |
|  | SINV | SINV (Sindbis virus) | Alphavirus | Western equine encephalitis (sindbis-like) |  |
|  | CCHFV | CCHFV (Crimean Congo Haemorrhagic fever virus) | Nairovirus | Crimean-Congo hemorrhagic fever |  |

**Table D – Circulation of arboviruses per area used in scoring system**All arboviruses circulating in specific geographic areas, according to Cleton et al (19,20), additional literature (21–43), and CDC and WHO circulation maps, were used to calculate chances of present pre-exposure antibodies in residence and travel areas of study participants. For arboviruses that only recently circulate, the reported year of the start of circulation in particular areas was taken into account. For calculating the effect of possible pre-exposure antibodies on the antibody cross-reactivity results, DENV and ZIKV were considered as the same serogroup based on their high antigenic similarities.

| Geographic region of specific arbovirus circulation | Residence and travel area (found in literature search) |
| --- | --- |
| 1 – Northern America | United states of America (USA)  Canada |
| 2 – Mexico, Central America and Caribbean | Mexico |
|  | Nicaragua  Guatemala |
|  | Puerto Rico |
|  | Cuba |
|  | Guadeloupe |
|  | Honduras |
|  | Aruba  Curaçao |
|  | Dominican Republic  Belize  Jamaica |
|  | Virgin Islands  Cape Verde  Cayman islands  St. Lucia  Martinique  Costa Rica  Panama  El Salvador  Barbados  Tobago |
| 3 - South America | Brazil |
|  | Colombia |
|  | Peru |
|  | Venezuela |
|  | Surinam  Chile  Ecuador  French Guyana  Paraguay  Bolivia  Guyana |
| 4 - North Africa | - |
| 5 - Sub-Saharan Africa | Nigeria |
|  | Kenya  Tanzania |
| 6 - Western and Central Asia | Israel  Kazakhstan |
| 7 - South-East, South and East Asia | Singapore |
|  | India |
|  | Myanmar |
|  | Sri Lanka |
|  | Thailand |
|  | Taiwan |
|  | Japan |
|  | China |
|  | Philippines |
|  | Maldives  Cambodia  Indonesia  Laos  Malaysia  Vietnam |
| 8 - Oceania | Micronesia |
|  | American Samoa  Papua New Guinea  Australia  Hawaii |
| 9 - Northern Europe | Finland |
| 10 - Southern Europe | Spain |
|  | Italy |
|  | Greece  Madeira |
| 11 - Central Europe | Austria |
|  | Switzerland |
|  | Germany |
|  | Lithuania |
| 12 - Western Europe | Netherlands |
|  | Belgium |
|  | UK  France |
| 13 - Eastern Europe (incl. Russia) | - |

**Table E – Classification of residence and travel areas per geographic area**
All residence and travel areas of the literature search were classified in determined geographic regions that have a similar composition of arbovirus circulation based on Cleton et al (19,20) and CDC and WHO circulation maps.

| Diagnostic specificity score | |
| --- | --- |
| Maximum score | 40 |
| Group A | 31 – 40 |
| Group B | 21 – 30 |
| Group C | 11 – 20 |
| Group D | 0 – 10 |

| Study size score | |
| --- | --- |
| Maximum score | 4 |
| Group A | 4 |
| Group B | 3 |
| Group C | 2 |
| Group D | 0 – 1 |

| Arbovirus background score | |
| --- | --- |
| Maximum score | 24 |
| Group A | 19 – 24 |
| Group B | 13 – 18 |
| Group C | 7 – 12 |
| Group D | 0 – 6 |

**Table F – Category groups of sum of points**Total sum of points of each category was divided into four quartiles (Group A, B, C and D) to be able to equally weigh and compare the total scores of the different categories.

| Combination | Arbovirus background score | Diagnostic specificity score | Study size score | Description | Number of studies | **Combination group** |
| --- | --- | --- | --- | --- | --- | --- |
| 1 | **A** | **A** | **A** | A, A, A | 0 | Group 1 |
| 2 | **B** | **A** | **A** | A, A, B | 0 | Group 2 |
| 3 | **A** | **B** | **A** | A, A, B | 0 | Group 2 |
| 4 | **A** | **A** | **B** | A, A, B | 0 | Group 2 |
| 5 | **C** | **A** | **A** | A, A, C | 0 | Group 3 |
| 6 | **A** | **C** | **A** | A, A, C | 0 | Group 3 |
| 7 | **A** | **A** | **C** | A, A, C | 0 | Group 3 |
| 8 | **A** | **B** | **B** | A, B, B | 0 | Group 3 |
| 9 | **B** | **A** | **B** | A, B, B | 0 | Group 3 |
| 10 | **B** | **B** | **A** | A, B, B | 0 | Group 3 |
| 11 | **D** | **A** | **A** | A, A, D | **8** | **Group 4** |
| 12 | **A** | **D** | **A** | A, A, D | 0 | Group 4 |
| 13 | **A** | **A** | **D** | A, A, D | 0 | Group 4 |
| 14 | **A** | **B** | **C** | A, B, C | 0 | Group 4 |
| 15 | **A** | **C** | **B** | A, B, C | 0 | Group 4 |
| 16 | **B** | **A** | **C** | A, B, C | **3** | **Group 4** |
| 17 | **C** | **A** | **B** | A, B, C | 0 | Group 4 |
| 18 | **B** | **C** | **A** | A, B, C | 0 | Group 4 |
| 19 | **C** | **B** | **A** | A, B, C | **1** | **Group 4** |
| 20 | **B** | **B** | **B** | B, B, B | 0 | Group 4 |
| 21 | **A** | **B** | **D** | A, B, D | 0 | Group 5 |
| 22 | **A** | **D** | **B** | A, B, D | 0 | Group 5 |
| 23 | **B** | **A** | **D** | A, B, D | **5** | **Group 5** |
| 24 | **D** | **A** | **B** | A, B, D | **11** | **Group 5** |
| 25 | **B** | **D** | **A** | A, B, D | 0 | Group 5 |
| 26 | **D** | **B** | **A** | A, B, D | **2** | **Group 5** |
| 27 | **A** | **C** | **C** | A, C, C | 0 | Group 5 |
| 28 | **C** | **A** | **C** | A, C, C | **50** | **Group 5** |
| 29 | **C** | **C** | **A** | A, C, C | 0 | Group 5 |
| 30 | **C** | **B** | **B** | B, B, C | **1** | **Group 5** |
| 31 | **B** | **C** | **B** | B, B, C | 0 | Group 5 |
| 32 | **B** | **B** | **C** | B, B, C | **1** | **Group 5** |
| 33 | **A** | **C** | **D** | A, C, D | 0 | Group 6 |
| 34 | **A** | **D** | **C** | A, C, D | 0 | Group 6 |
| 35 | **C** | **A** | **D** | A, C, D | **40** | **Group 6** |
| 36 | **D** | **A** | **C** | A, C, D | **335** | **Group 6** |
| 37 | **C** | **D** | **A** | A, C, D | 0 | Group 6 |
| 38 | **D** | **C** | **A** | A, C, D | **4** | **Group 6** |
| 39 | **D** | **B** | **B** | B, B, D | **2** | **Group 6** |
| 40 | **B** | **D** | **B** | B, B, D | 0 | Group 6 |
| 41 | **B** | **B** | **D** | B, B, D | 0 | Group 6 |
| 42 | **B** | **C** | **C** | B, C, C | 0 | Group 6 |
| 43 | **C** | **B** | **C** | B, C, C | **11** | **Group 6** |
| 44 | **C** | **C** | **B** | B, C, C | 0 | Group 6 |
| 45 | **A** | **D** | **D** | A, D, D | 0 | Group 7 |
| 46 | **D** | **A** | **D** | A, D, D | **284** | **Group 7** |
| 47 | **D** | **D** | **A** | A, D, D | **7** | **Group 7** |
| 48 | **B** | **D** | **C** | B, C, D | 0 | Group 7 |
| 49 | **B** | **C** | **D** | B, C, D | 0 | Group 7 |
| 50 | **D** | **B** | **C** | B, C, D | **75** | **Group 7** |
| 51 | **C** | **B** | **D** | B, C, D | **5** | **Group 7** |
| 52 | **D** | **C** | **B** | B, C, D | **4** | **Group 7** |
| 53 | **C** | **D** | **B** | B, C, D | 0 | Group 7 |
| 54 | **C** | **C** | **C** | C, C, C | **23** | **Group 7** |
| 55 | **B** | **D** | **D** | B, D, D | 0 | Group 8 |
| 56 | **D** | **B** | **D** | B, D, D | **39** | **Group 8** |
| 57 | **D** | **D** | **B** | B, D, D | **3** | **Group 8** |
| 58 | **D** | **C** | **C** | C, C, D | **26** | **Group 8** |
| 59 | **C** | **D** | **C** | C, C, D | **13** | **Group 8** |
| 60 | **C** | **C** | **D** | C, C, D | **0** | Group 8 |
| 61 | **C** | **D** | **D** | C, D, D | **12** | **Group 9** |
| 62 | **D** | **C** | **D** | C, D, D | **23** | **Group 9** |
| 63 | **D** | **D** | **C** | C, D, D | **48** | **Group 9** |
| 64 | **D** | **D** | **D** | D, D, D | **46** | **Group 10** |

**Table G – Overview combinations A, B, C and D of the three categories scoring reliability**
The table shows an overview of all the possible combinations and the found combinations (A, B, C and D) of the three main categories scoring reliability of the included studies (N = 1082). The table is ordered by showing the best possible reliable combination at the top of the table and the least possible reliable combination at the bottom of the table.

**Table H – Overview of total reliability groups** **based on category combination score**

Total reliability groups were defined per category combination score of A, B, C and D for included studies (N = 1082). Total reliability group 1 is most reliable, whereas total reliability group 5 is the least reliable. Number of studies per total reliability group can be seen in the last column

| Combination (in all different orders) | **Combination group** | **Number of studies** | **Total reliability group** | **Number of studies** |
| --- | --- | --- | --- | --- |
| A - A - A | **Group 1** | **0** | **Group 1 (Most reliable)** | **0** |
| A - A - B | **Group 2** | **0** |  |  |
| A - A - C  A - B - B | **Group 3** | **0** | **Group 2 (Highly reliable)** | **12** |
| A - A - D  A - B - C  B - B - B | **Group 4** | **12** |  |  |
| A - B - D  A - C - C  B - B - C | **Group 5** | **70** | **Group 3 (Medium reliable)** | **462** |
| A - C - D  B - B - D  B - C - C | **Group 6** | **392** |  |  |
| A - D - D  B - C - D  C - C - C | **Group 7** | **398** | **Group 4 (Low reliable)** | **479** |
| B - D - D  C - C - D | **Group 8** | **81** |  |  |
| C - D - D | **Group 9** | **83** | **Group 5 (Least reliable)** | **129** |
| D - D - D | **Group 10** | **46** |  |  |

| **Variable** | **Description** | **Exemplar data** | **Recommendation** | **To fill in** |
| --- | --- | --- | --- | --- |
| **Virus of exposure** | The virus to which the individuals included in the study are exposed (infection or vaccination) | *e.g., ZIKV (infection)*  *e.g., YFV (vaccination)* |  |  |
| **Residence area** (Country, state, city, district) | The country and additional information about the residence of the individuals included in the study | *e.g., Brazil, Pernambuco, Recife, Boa Viagem* | *For the most reliable assessment of antibody cross-reactivity, preferably a country that is least likely biased from possible previous arbovirus exposures with the same virus as or a virus closely related to the virus tested for cross-reactivity of antibodies induced by the virus of exposure.* |  |
| **Travel area of travellers** (Country, state, city, district) | The area of acquired infection of travellers included in the study | *e.g., Indonesia, Java, Jakarta, Cengkareng* | *For the most reliable assessment of antibody cross-reactivity, preferably a country that is least likely biased from possible previous arbovirus exposures with the same virus as or a virus closely related to the virus tested for cross-reactivity of antibodies induced by the virus of exposure.* |  |
| **Prior travel history** (non-travellers & travellers) (Country, state, city, district) | The prior travel history of the individuals included in the study | *e.g., No travel in the previous 5 years or “* | *For the most reliable assessment of antibody cross-reactivity, preferably no (recent) prior travel to areas endemic for viruses that include the same virus as or a virus closely related to the virus tested for cross-reactivity of antibodies induced by the virus of exposure.* |  |
| **Prior arbovirus vaccination history** | The prior arbovirus vaccination history of individuals included in the study | *e.g., YFV and JEV vaccination 5 years ago or no prior vaccination with either TBEV, YFV or JEV* | *Include the year of vaccination if possible. Also report if the individuals did not have any prior relevant arbovirus vaccinations, since this minimizes the chance of bias from pre-exposure antibodies on the antibody cross-reactivity results.* |  |
| **Age** | The age of the studied individuals (children, adults or both with the range of ages) | *e.g., Children (3-16)*  *e.g., Adults (26-54)* | *Recommended to study children in case of an infection area that is endemic to the same virus or viruses closely related to the virus tested for cross-reactivity, to minimize the chance of present pre-exposure antibodies that may bias the antibody cross-reactivity results.* |  |
| **Prior arbovirus infection history** | Evidence of no or prior infection history by serological or molecular methods | *e.g., DENV-naïve*  *e.g., Secondary ZIKV* | *Recommended to test for (IgG) antibodies around day 0 since symptom onset (or earlier) to define the infection status (naïve, primary, secondary, …) or, if possible, follow the individuals in time to detect possible infections prior to the recent infection by molecular methods.* |  |

**Table I - A minimum standard for metadata regarding the studied individuals**

| **Variable** | **Description** | **Exemplar data** | **Recommendation** | **To fill in** |
| --- | --- | --- | --- | --- |
| ***Methods used for confirmation of arbovirus exposure*** | | | | |
| **Methods used** | The molecular and/or serological methods used to confirm the virus exposure of the studied individuals | *e.g., PCR*  *e.g., PCR and VNT*  *e.g., ELISA and VNT* | *Recommended to use molecular methods since these provide the highest quality of evidence. If using serological methods, use at least two methods to confirm the results (preferably confirmation by VNT). Also mention details regarding the assay format, e.g., inhibition ELISA or plaque reduction neutralization test.* |  |
| **Cut-off used** | The cut-off used to determine whether the test result is positive or negative | *e.g., IFA Cut-off IgM: ≥ 1:10 and IgG: ≥ 1:100*  *e.g., ELISA cut-off of ≥ 1.1* | *Recommended to use standardized cut-offs to be able to compare results from multiple different studies.* |  |
| **Type of antibody tested**  *(serological methods only)* | The type of antibody tested in serological assays to diagnose individuals | *e.g., IgG*  *e.g., IgM* |  |  |
| **Antigen tested** *(serological methods only)* | The type of antigen used to confirm the virus of exposure | *e.g., NS1*  *e.g., E protein*  *e.g., EDIII protein*  *e.g., produced in mammalian HEK293 cells* | *Recommended to use an antigen as specific as possible to minimize antibody cross-reactivity. Also provide specifics (e.g., source, batch)* |  |
| **Case definition** | Case definition used to determine the virus of exposure | *e.g., seroconversion or 4-fold increase using paired sera (with negative results or lower increasing titers for closely related viruses that likely cross-react)* | *For the most reliable assessment of the virus of infection, use paired sera from both the acute and convalescent phase, to be able to look at seroconversion and/or 4-fold increase. Preferably, compare this with the titers of closely related viruses that likely cross-react for the most accurate differentiation.* |  |
| **Timepoints of serum samples** | The timing of sera sampling used for the case definition | *e.g., Single serum after 10 dpso or paired sera before 7 and after 14 dpso* | *Either use a single serum after 10 days post symptom onset to be able to detect the induced antibody response by the recent infection, or preferably paired sera before day 7 and after day 14 since symptom onset to be able to detect seroconversion and/or 4-fold increase over time. Recommended to report this for all studied individuals.* |  |
| **Controls used** | The negative and positive controls used | e*.g., positive VNT and PCR confirmed ZIKV sera* | *Recommended to use standardized control panels to be able to compare results from multiple different studies.* |  |
| **Raw data** | The raw data output of the method(s) used | e.g., IgG titer values (for instance a titer of 120) | *Recommended to provide the raw data values of the method(s) used. In case of testing for multiple (closely-related) viruses, also provide the values of these viruses.* |  |
| ***Methods used to determine the arbovirus infection history*** | | | | |
| **Methods used** | The molecular and/or serological methods used to determine the infection history | *e.g., PCR*  *e.g., PCR and VNT*  *e.g., ELISA and VNT* | *Recommended to also mention details regarding the assay format, e.g., inhibition ELISA or plaque reduction neutralization test.* |  |
| **Cut-off used** | The cut-off used to determine whether the test result is positive or negative | *e.g., IFA Cut-off IgM: ≥ 1:10 and IgG: ≥ 1:100*  *e.g., ELISA cut-off of ≥ 1.1* | *Recommended to use standardized cut-offs to be able to compare results from multiple different studies.* |  |
| **Type of antibody tested**  *(serological methods only)* | The type of antibody tested in serological assays | *e.g., IgG*  *e.g., IgM* | *Recommended to measure IgG antibodies or IgM and IgG antibodies and look at the IgM/IgG ratio, to define whether individuals have experienced previous arbovirus infections or not.* |  |
| **Antigen tested** *(serological methods only)* | The type of antigen used | *e.g., NS1*  *e.g., E protein*  *e.g., EDIII protein*  *e.g., produced in mammalian HEK293 cells* | *Recommended to use an antigen as specific as possible to minimize antibody cross-reactivity. Also provide specifics (e.g., source, batch)* |  |
| **Definition of infection history status** | Interpretation of test results with regards to the infection history status | *e.g., IgM/IgG ratios ≥1.2 are indicative of primary infections and ratios <1.2 of secondary infections*  *e.g., Positive IgG indicates prior infection and negative IgG no prior infections* |  |  |
| **Timepoints of serum samples** | The sera sampling timepoints used to determine the infection history | *e.g., Day 0-2 since symptom onset* | *Recommended to measure antibodies around day 0 since symptom onset (or earlier) to correctly define the prior infection history.* |  |
| **Controls used** | The negative and positive controls used | e*.g., positive VNT and PCR confirmed ZIKV sera* | *Recommended to use standardized control panels to be able to compare results from multiple different studies.* |  |
| **Raw data** | The raw data output of the method(s) used | e.g., IgG titer values (for instance a titer of 120) | *Recommended to provide the raw data values of the method(s) used. In case of testing for multiple (closely-related) viruses, also provide the values of these viruses.* |  |
| ***Methods used to test antibody cross-reactivity*** | | | | |
| **Panel of viruses included to test for antibody cross-reactivity** | The virus to which antibody reactivity is tested by antibodies induced by the virus of recent confirmed exposure | *e.g., WNV* |  |  |
| **Serological method used** | Serological test format used for cross-reactivity testing | *e.g., ELISA*  *e.g., VNT* | *Preferably also mention other details like inhibition ELISA.* |  |
| **Cut-off used** | The cut-off used to determine whether the test result is positive or negative | *e.g., IFA Cut-off IgM: ≥ 1:10 and IgG: ≥ 1:100*  *e.g., WNV/SLEV ratio of ≥3.0 was defined as a WNV infection* | *Recommended to use standardized cut-offs to be able to compare results from multiple different studies.* |  |
| **Type of antibody tested** | Type of antibody tested in cross-reactivity method | *e.g., IgM*  *e.g., IgG* |  |  |
| **Antigen tested** | The type of antigen used | *e.g., NS1*  *e.g., E protein*  *e.g., EDIII protein*  *e.g., whole virus*  *e.g., produced in mammalian HEK293 cells* | *Recommended to provide specifics (e.g., source, batch)* |  |
| **Timepoints of serum samples** | The sera sampling timepoints of the sera used to test antibody cross-reactivity | *e.g., Acute sera 2-6 dpso*  *e.g., Convalescent sera 21-40 dpso* |  |  |
| **Controls used** | The negative and positive controls used | *e.g., positive confirmed ZIKV sera* | *Recommended to use standardized control panels to be able to compare results from multiple different studies.* |  |
| **Raw data** | The raw data output of the method(s) used | e.g., IgG titer values (for instance a titer of 120) | *Recommended to provide the raw data values of the method(s) used.* |  |
| **# Total confirmed positives** | The number of total confirmed exposed individuals used to test antibody cross-reactivity | *e.g., 124* | *The number of total confirmed positive individuals that preferably also show antibody reactivity to the virus of confirmation with the same type of antibody as used in the method to test antibody cross-reactivity. For the best comparison, if possible, use the number of confirmed positives that also show reactivity to the virus of confirmation in the same method as used to test antibody cross-reactivity.* |  |
| **# Confirmed positives that show reactivity to other antigens** | The number of confirmed exposed individuals that show reactivity to both the virus of infection and the virus tested for cross-reactivity | *e.g., 31* | *As above* |  |
| **Definition of antibody cross-reactivity (antibody cross-reactivity %)** | The number of positives in a heterologous serological assay (testing for a different virus than the virus of exposure) divided by the total number of confirmed exposed individuals (# Confirmed positives that show reactivity to other antigens / # Total confirmed positives * 100) | *e.g., 31/124 * 100 = 25%* | *See above. The antibody cross-reactivity % should reflect how often samples of virus X also show antibody reactivity to virus Y with the same type of antibody and preferably also in the same type of test.* |  |

**Table J - A minimum standard for metadata regarding the methods used to confirm the exposure, determine the prior infection history and test for antibody cross-reactivity**

**Supplemental figures**


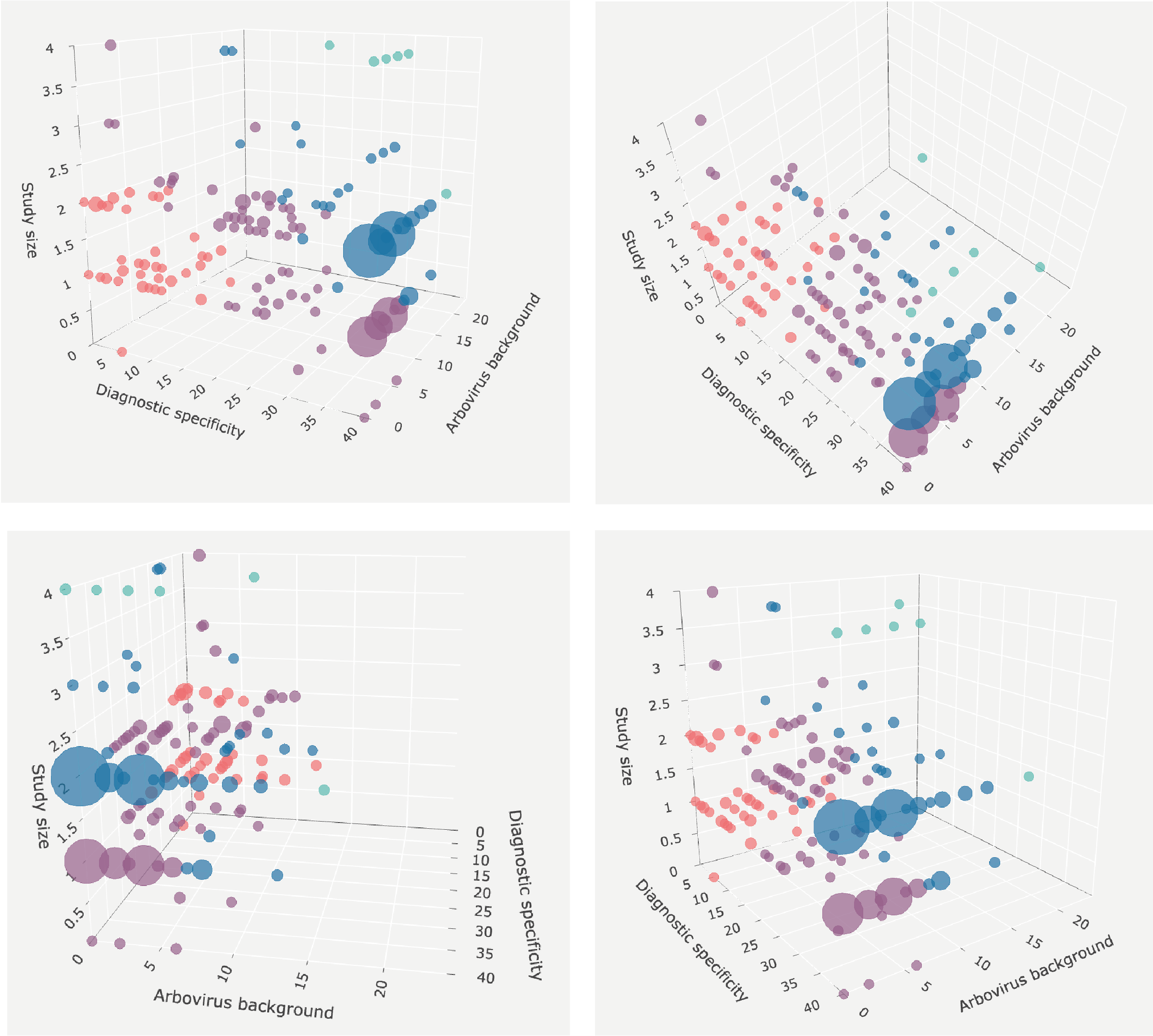


**Fig A – Three-dimensional plots of category scores and total reliability groups**

Scores for all three categories (study size, diagnostic specificity and arbovirus background) in three-dimensional plots (N= 1082). Total reliability groups are depicted in colours ranging from group 1 (highest) to group 5 (lowest) (yellow, turquoise, dark blue, purple and pink, respectively). Frequency of a specific score is shown by size.

**References**

1. CDC. Human Laboratory Diagnosis and Testing | Mosquitoes | CDC [Internet]. [cited 2022 Nov 29]. Available from: https://www.cdc.gov/mosquitoes/guidelines/west-nile/laboratory-diagnosis-testing/index.html

2. PAHO and WHO. Tool for the diagnosis and care of patients with suspected arboviral diseases. 2017;

3. CDC. Zika Virus Disease and Zika Virus Infection 2016 Case Definition [Internet]. [cited 2022 Mar 11]. Available from: https://ndc.services.cdc.gov/case-definitions/zika-virus-disease-and-zika-virus-infection-2016-06-01/

4. CDC. Arboviral Diseases, Neuroinvasive and Non-neuroinvasive 2015 Case Definition [Internet]. [cited 2022 Mar 11]. Available from: https://ndc.services.cdc.gov/case-definitions/arboviral-diseases-neuroinvasive-and-non-neuroinvasive-2015/

5. CDC. Surveillance Case Definitions for Current and Historical Conditions [Internet]. [cited 2022 Mar 11]. Available from: https://ndc.services.cdc.gov/

6. Musso D, Desprès P. Serological Diagnosis of Flavivirus-Associated Human Infections. Diagnostics 2020, Vol 10, Page 302 [Internet]. 2020 May 14 [cited 2022 Dec 1];10(5):302. Available from: https://www.mdpi.com/2075-4418/10/5/302/htm

7. Chan KR, Ismail AA, Thergarajan G, Raju CS, Yam HC, Rishya M, et al. Serological cross-reactivity among common flaviviruses. Front Cell Infect Microbiol [Internet]. 2022 Sep 15 [cited 2023 Aug 2];12. Available from: /pmc/articles/PMC9519894/

8. Plotkin SA. Correlates of Protection Induced by Vaccination. Clin Vaccine Immunol [Internet]. 2010 [cited 2023 Aug 2];17(7):1055. Available from: /pmc/articles/PMC2897268/

9. Guy B, Guirakhoo F, Barban V, Higgs S, Monath TP, Lang J. Preclinical and clinical development of YFV 17D-based chimeric vaccines against dengue, West Nile and Japanese encephalitis viruses. Vaccine [Internet]. 2010 Jan 8 [cited 2023 Aug 2];28(3):632–49. Available from: https://pubmed.ncbi.nlm.nih.gov/19808029/

10. Thomas SJ, Nisalak A, Anderson KB, Libraty DH, Kalayanarooj S, Vaughn DW, et al. Dengue Plaque Reduction Neutralization Test (PRNT) in Primary and Secondary Dengue Virus Infections: How Alterations in Assay Conditions Impact Performance. Am J Trop Med Hyg [Internet]. 2009 Nov [cited 2023 Aug 2];81(5):825. Available from: /pmc/articles/PMC2835862/

11. CDC. Dengue Virus Infections 2015 Case Definition | CDC [Internet]. [cited 2022 Nov 29]. Available from: https://ndc.services.cdc.gov/case-definitions/dengue-virus-infections-2015/

12. Balmaseda A, Stettler K, Medialdea-Carrera R, Collado D, Jin X, Zambrana JV, et al. Antibody-based assay discriminates Zika virus infection from other flaviviruses. Proceedings of the National Academy of Sciences. 2017;201704984.

13. Mora-Cárdenas E, Aloise C, Faoro V, Knap Gašper N, Korva M, Caracciolo I, et al. Comparative specificity and sensitivity of NS1-based serological assays for the detection of flavivirus immune response. Asgari S, editor. PLoS Negl Trop Dis [Internet]. 2020 Jan 29 [cited 2020 Jul 20];14(1):e0008039. Available from: https://dx.plos.org/10.1371/journal.pntd.0008039

14. Stettler K, Beltramello M, Espinosa DA, Graham V, Cassotta A, Bianchi S, et al. Specificity, cross-reactivity, and function of antibodies elicited by Zika virus infection. Science (1979) [Internet]. 2016 Aug 19 [cited 2020 Jul 20];353(6301):823–6. Available from: http://science.sciencemag.org/

15. Lima M da RQ, Chouin-Carneiro T, Azeredo E, Barbosa LS, Souza TMA, Silva JBC da, et al. The inability of a dengue NS1 ELISA to detect Zika infections. Sci Rep [Internet]. 2019 Dec 1 [cited 2020 Jul 20];9(1):1–7. Available from: www.nature.com/scientificreports

16. Cleton NB, van Maanen K, Bergervoet SA, Bon N, Beck C, Godeke GJ, et al. A Serological Protein Microarray for Detection of Multiple Cross-Reactive Flavivirus Infections in Horses for Veterinary and Public Health Surveillance. Transbound Emerg Dis [Internet]. 2017 Dec 1 [cited 2020 Jul 20];64(6):1801–12. Available from: https://pubmed.ncbi.nlm.nih.gov/27633257/

17. Cleton NB, Godeke GJ, Reimerink J, Beersma MF, Doorn HR van, Franco L, et al. Spot the Difference—Development of a Syndrome Based Protein Microarray for Specific Serological Detection of Multiple Flavivirus Infections in Travelers. PLoS Negl Trop Dis. 2015;9(3):1–17.

18. Balmaseda A, Zambrana JV, Collado D, García N, Saborío S, Elizondo D, et al. Comparison of four serological methods and two reverse transcription-PCR assays for diagnosis and surveillance of Zika virus infection. J Clin Microbiol [Internet]. 2018 Mar 1 [cited 2020 Aug 13];56(3). Available from: /pmc/articles/PMC5824050/?report=abstract

19. Cleton NB, Reusken CBEM, Wagenaar JFP, van der Vaart EE, Reimerink J, van der Eijk AA, et al. Syndromic Approach to Arboviral Diagnostics for Global Travelers as a Basis for Infectious Disease Surveillance. Franco-Paredes C, editor. PLoS Negl Trop Dis [Internet]. 2015 Sep 15 [cited 2020 Jul 20];9(9):e0004073. Available from: https://dx.plos.org/10.1371/journal.pntd.0004073

20. Cleton N, Koopmans M, Reimerink J, Godeke GJ, Reusken C. Come fly with me: Review of clinically important arboviruses for global travelers. Vol. 55, Journal of Clinical Virology. Elsevier; 2012. p. 191–203.

21. Pierson TC, Diamond MS. The continued threat of emerging flaviviruses. Nature Microbiology 2020 5:6 [Internet]. 2020 May 4 [cited 2021 Jul 15];5(6):796–812. Available from: https://www.nature.com/articles/s41564-020-0714-0

22. Rückert C, Weger-Lucarelli J, Garcia-Luna SM, Young MC, Byas AD, Murrieta RA, et al. Impact of simultaneous exposure to arboviruses on infection and transmission by Aedes aegypti mosquitoes. Nature Communications 2017 8:1 [Internet]. 2017 May 19 [cited 2021 Jul 29];8(1):1–9. Available from: https://www.nature.com/articles/ncomms15412

23. GUBLER DJ. Human Arbovirus Infections Worldwide. Ann N Y Acad Sci [Internet]. 2006 Dec 1 [cited 2021 Jul 30];951(1):13–24. Available from: https://nyaspubs.onlinelibrary.wiley.com/doi/full/10.1111/j.1749-6632.2001.tb02681.x

24. Añez G, Rios M. Dengue in the United States of America: A Worsening Scenario? Biomed Res Int [Internet]. 2013 [cited 2023 Aug 1];2013:13. Available from: /pmc/articles/PMC3705843/

25. Fischer M, Staples JE. Chikungunya Virus Spreads in the Americas — Caribbean and South America, 2013–2014. Morbidity and Mortality Weekly Report [Internet]. 2014 Jun 6 [cited 2023 Aug 2];63(22):500. Available from: /pmc/articles/PMC5779358/

26. Fauci AS, Morens DM. Zika Virus in the Americas — Yet Another Arbovirus Threat. New England Journal of Medicine [Internet]. 2016 Feb 18 [cited 2023 Aug 2];374(7):601–4. Available from: https://www.nejm.org/doi/10.1056/NEJMp1600297

27. Notes from the Field: Transmission of Chikungunya Virus in the Continental United States — Florida, 2014 [Internet]. [cited 2023 Aug 2]. Available from: https://www.cdc.gov/mmwr/preview/mmwrhtml/mm6348a4.htm

28. Cunha MS, Costa PAG, Correa IA, de Souza MRM, Calil PT, da Silva GPD, et al. Chikungunya Virus: An Emergent Arbovirus to the South American Continent and a Continuous Threat to the World. Front Microbiol. 2020 Jun 26;11:532919.

29. Akinsulie OC, Adesola RO, Bakre A, Adebowale OO, Adeleke R, Ogunleye SC, et al. Usutu virus: An emerging flavivirus with potential threat to public health in Africa: Nigeria as a case study. Front Vet Sci. 2023 Feb 16;10:1115501.

30. Plourde AR, Bloch EM. A Literature Review of Zika Virus. Emerg Infect Dis [Internet]. 2016 Jul 1 [cited 2023 Aug 2];22(7):1185. Available from: /pmc/articles/PMC4918175/

31. Duffy MR, Chen TH, Hancock WT, Powers AM, Kool JL, Lanciotti RS, et al. Zika virus outbreak on Yap Island, Federated States of Micronesia. New England Journal of Medicine. 2009 Jun 11;360(24):2536–43.

32. Chen X, Li F, Yin Q, Liu W, Fu S, He Y, et al. Epidemiology of tick-borne encephalitis in China, 2007- 2018. PLoS One [Internet]. 2019 Dec 1 [cited 2023 Aug 2];14(12). Available from: /pmc/articles/PMC6932775/

33. Horwood P, Bande G, Dagina R, Guillaumot L, Aaskov J, Pavlin B. The threat of chikungunya in Oceania. Western Pac Surveill Response J [Internet]. 2013 Apr 1 [cited 2023 Aug 2];4(2):8. Available from: /pmc/articles/PMC3762969/

34. Amraoui F, Failloux AB. Chikungunya: an unexpected emergence in Europe. Curr Opin Virol [Internet]. 2016 Dec 1 [cited 2023 Aug 2];21:146–50. Available from: https://pubmed.ncbi.nlm.nih.gov/27771517/

35. Rezza G, Nicoletti L, Angelini R, Romi R, Finarelli A, Panning M, et al. Infection with chikungunya virus in Italy: an outbreak in a temperate region. Lancet [Internet]. 2007 Dec 1 [cited 2023 Aug 2];370(9602):1840–6. Available from: http://www.thelancet.com/article/S0140673607617796/fulltext

36. Schaffner F, Mathis A. Dengue and dengue vectors in the WHO European region: Past, present, and scenarios for the future. Lancet Infect Dis [Internet]. 2014 Dec 1 [cited 2023 Aug 2];14(12):1271–80. Available from: http://www.thelancet.com/article/S1473309914708345/fulltext

37. Sousa CA, Clairouin M, Seixas G, Viveiros B, Novo MT, Silva AC, et al. Ongoing outbreak of dengue type 1 in the Autonomous Region of Madeira, Portugal: Preliminary report. Eurosurveillance [Internet]. 2012 Dec 6 [cited 2023 Aug 2];17(49):20333. Available from: https://www.eurosurveillance.org/content/10.2807/ese.17.49.20333-en

38. Autochthonous vectorial transmission of dengue virus in mainland EU/EEA, 2010-present [Internet]. [cited 2023 Aug 2]. Available from: https://www.ecdc.europa.eu/en/all-topics-z/dengue/surveillance-and-disease-data/autochthonous-transmission-dengue-virus-eueea

39. Vilibic-Cavlek T, Petrovic T, Savic V, Barbic L, Tabain I, Stevanovic V, et al. Epidemiology of Usutu Virus: The European Scenario. Pathogens [Internet]. 2020 Sep 1 [cited 2023 Aug 2];9(9):1–19. Available from: /pmc/articles/PMC7560012/

40. Young JJ, Haussig JM, Aberle SW, Pervanidou D, Riccardo F, Sekulić N, et al. Epidemiology of human West Nile virus infections in the European Union and European Union enlargement countries, 2010 to 2018. Eurosurveillance [Internet]. 2021 May 5 [cited 2023 Aug 2];26(19):1. Available from: /pmc/articles/PMC8120798/

41. Cochet A, Calba C, Jourdain F, Grard G, Durand GA, Guinard A, et al. Autochthonous dengue in mainland France, 2022: geographical extension and incidence increase. Eurosurveillance [Internet]. 2022 Nov 11 [cited 2023 Aug 2];27(44):1. Available from: /pmc/articles/PMC9635021/

42. Jeffries CL, Mansfield KL, Phipps LP, Wakeley PR, Mearns R, Schock A, et al. Louping ill virus: an endemic tick-borne disease of Great Britain. J Gen Virol [Internet]. 2014 May 5 [cited 2023 Aug 2];95(Pt 5):1005. Available from: /pmc/articles/PMC4811648/

43. Bakonyi T, Haussig JM. West Nile virus keeps on moving up in Europe. Eurosurveillance [Internet]. 2020 Nov 11 [cited 2023 Aug 2];25(46). Available from: /pmc/articles/PMC7678036/

44. Power GM, Vaughan AM, Qiao L, Clemente NS, Pescarini JM, Paixão ES, et al. Socioeconomic risk markers of arthropod-borne virus (arbovirus) infections: a systematic literature review and meta-analysis. BMJ Glob Health [Internet]. 2022 Apr 1 [cited 2022 Nov 29];7(4):e007735. Available from: https://gh.bmj.com/content/7/4/e007735

45. Griffin I, Martin SW, Fischer M, Chambers T V, Kosoy O, Falise A, et al. Zika virus igm detection and neutralizing antibody profiles 12–19 months after illness onset. Emerg Infect Dis [Internet]. 2019;25(2):299–303. Available from: http://www.embase.com/search/results?subaction=viewrecord&from=export&id=L2001498360

46. Gordon A, Gresh L, Ojeda S, Katzelnick LC, Sanchez N, Mercado JC, et al. Prior dengue virus infection and risk of Zika: A pediatric cohort in Nicaragua. PLoS Med [Internet]. 2019;16(1). Available from: http://www.embase.com/search/results?subaction=viewrecord&from=export&id=L2001519364

47. Van den Bossche D, Michiels J, Cnops L, Foque N, Meersman K, Huits R, et al. Challenges in diagnosing Zika—experiences from a reference laboratory n a non-endemic setting. Eur J Clin Microbiol Infect Dis [Internet]. 2019; Available from: http://www.embase.com/search/results?subaction=viewrecord&from=export&id=L626156227

48. Jääskeläinen AJ, Korhonen EM, Huhtamo E, Lappalainen M, Vapalahti O, Kallio-Kokko H. Validation of serological and molecular methods for diagnosis of zika virus infections. J Virol Methods. 2019 Jan 1;263:68–74.

49. De Ory F, Sánchez-Seco MP, Vázquez A, Montero MD, Sulleiro E, Martínez MJ, et al. Comparative evaluation of indirect immunofluorescence and NS-1-based ELISA to determine zika virus-specific IgM. Viruses [Internet]. 2018;10(7). Available from: http://www.embase.com/search/results?subaction=viewrecord&from=export&id=L623142173

50. Montoya M, Collins M, Dejnirattisai W, Katzelnick LC, Puerta-Guardo H, Jadi R, et al. Longitudinal analysis of antibody cross-neutralization following zika virus and dengue virus infection in Asia and the Americas. J INFECT DIS [Internet]. 2018;218(4):536–45. Available from: http://www.embase.com/search/results?subaction=viewrecord&from=export&id=L623317295

51. João EC, Ferreira ODC, Gouvêa MI, Teixeira MDLB, Tanuri A, Higa LM, et al. Pregnant women co-infected with HIV and zika: Outcomes and birth defects in infants according to maternal symptomatology. PLoS One [Internet]. 2018;13(7). Available from: http://www.embase.com/search/results?subaction=viewrecord&from=export&id=L622895327

52. Premkumar L, Collins M, Graham S, Liou GJA, Lopez CA, Jadi R, et al. Development of envelope protein antigens to serologically differentiate zika virus infection from dengue virus infection. J Clin Microbiol. 2018 Mar 1;56(3).

53. Lindsey NP, Staples JE, Powell K, Rabe IB, Fischer M, Powers AM, et al. Ability to serologically confirm recent Zika virus infection in areas with varying past incidence of dengue virus infection in the United States and U.S. Territories in 2016. J CLIN MICROBIOL [Internet]. 2018;56(1). Available from: http://www.embase.com/search/results?subaction=viewrecord&from=export&id=L620138384

54. Lai L, Rouphael N, Xu Y, Natrajan MS, Beck A, Hart M, et al. Innate, T-, and B-Cell Responses in Acute Human Zika Patients. CLIN INFECT DIS [Internet]. 2018;66(1):1–10. Available from: http://www.embase.com/search/results?subaction=viewrecord&from=export&id=L620253977

55. Magalhaes T, Braga C, Cordeiro MT, Oliveira ALS, Castanha PMS, Maciel APR, et al. Zika virus displacement by a chikungunya outbreak in Recife, Brazil. PLoS Negl Trop Dis [Internet]. 2017;11(11). Available from: http://www.embase.com/search/results?subaction=viewrecord&from=export&id=L619495176

56. Lai L, Rouphael N, Xu Y, Natrajan MS, Beck A, Hart M, et al. Innate, T-, and B-Cell Responses in Acute Human Zika Patients. Clin Infect Dis. 2018 Jan 1;66(1):1–10.

57. Felix AC, Souza NCS, Figueiredo WM, Costa AA, Inenami M, da Silva RMG, et al. Cross reactivity of commercial anti-dengue immunoassays in patients with acute Zika virus infection. J MED VIROL [Internet]. 2017;89(8):1477–9. Available from: http://www.embase.com/search/results?subaction=viewrecord&from=export&id=L614676297

58. van Meer MPA, Mögling R, Klaasse J, Chandler FD, Pas SD, van der Eijk AA, et al. Re-evaluation of routine dengue virus serology in travelers in the era of Zika virus emergence. J Clin Virol [Internet]. 2017;92:25–31. Available from: http://www.embase.com/search/results?subaction=viewrecord&from=export&id=L616109111

59. Priyamvada L, Quicke KM, Hudson WH, Onlamoon N, Sewatanon J, Edupuganti S, et al. Human antibody responses after dengue virus infection are highly cross-reactive to Zika virus. Proceedings of the National Academy of Sciences [Internet]. 2016;113(28):7852–7. Available from: http://www.pnas.org/lookup/doi/10.1073/pnas.1607931113

60. Singh KP, Mishra G, Jain P, Pandey N, Nagar R, Gupta S, et al. Co-positivity of anti-dengue virus and anti-Japanese encephalitis virus IgM in endemic area: Co-infection or cross reactivity? Asian Pac J Trop Med [Internet]. 2014;7(2):124–9. Available from: http://www.embase.com/search/results?subaction=viewrecord&from=export&id=L372201006

61. Toh YX, Gan V, Balakrishnan T, Zuest R, Poidinger M, Wilson S, et al. Dengue serotype cross-reactive, anti-E protein antibodies confound specific immune memory for 1 year after infection. Front Immunol [Internet]. 2014;5(AUG). Available from: http://www.embase.com/search/results?subaction=viewrecord&from=export&id=L373813393

62. Pierro A, Landini MP, Gaibani P, Rossini G, Vocale C, Finarelli AC, et al. A model of laboratory surveillance for neuro-arbovirosis applied during 2012 in the Emilia-Romagna region, Italy. Clin Microbiol Infect [Internet]. 2014;20(7):672–7. Available from: http://www.embase.com/search/results?subaction=viewrecord&from=export&id=L373624416

63. Ngwe Tun MM, Thant KZ, Inoue S, Nabeshima T, Aoki K, Kyaw AK, et al. Detection of East/Central/South African genotype of chikungunya virus in Myanmar, 2010. Emerg Infect Dis [Internet]. 2014;20(8):1378–81. Available from: http://www.embase.com/search/results?subaction=viewrecord&from=export&id=L373678533

64. Maldaner FR, Aragão FJL, Dos Santos FB, Franco OL, Da Rocha Queiroz Lima M, De Oliveira Resende R, et al. Dengue virus tetra-epitope peptide expressed in lettuce chloroplasts for potential use in dengue diagnosis. Appl Microbiol Biotechnol [Internet]. 2013;97(13):5721–9. Available from: http://www.embase.com/search/results?subaction=viewrecord&from=export&id=L52553004

65. Vratskikh O, Stiasny K, Zlatkovic J, Tsouchnikas G, Jarmer J, Karrer U, et al. Dissection of Antibody Specificities Induced by Yellow Fever Vaccination. PLoS Pathog [Internet]. 2013;9(6). Available from: http://www.embase.com/search/results?subaction=viewrecord&from=export&id=L369208267

66. Marrero-Santos KM, Beltrán M, Carrión-Lebrón J, Sanchez-Vegas C, Hamer DH, Barnett ED, et al. Optimization of the cutoff value for a commercial anti-dengue virus IgG immunoassay. Clin Vaccine Immunol [Internet]. 2013;20(3):358–62. Available from: http://www.embase.com/search/results?subaction=viewrecord&from=export&id=L368530496

67. Puschnik A, Lau L, Cromwell EA, Balmaseda A, Zompi S, Harris E. Correlation between Dengue-Specific Neutralizing Antibodies and Serum Avidity in Primary and Secondary Dengue Virus 3 Natural Infections in Humans. PLoS Negl Trop Dis [Internet]. 2013;7(6). Available from: http://www.embase.com/search/results?subaction=viewrecord&from=export&id=L369208216

68. Garcia-Bates TM, Cordeiro MT, Nascimento EJM, Smith AP, De Melo KMS, McBurney SP, et al. Association between magnitude of the virus-specific plasmablast response and disease severity in dengue patients. J IMMUNOL [Internet]. 2013;190(1):80–7. Available from: http://www.embase.com/search/results?subaction=viewrecord&from=export&id=L368017024

69. Lai CY, Williams KL, Wu YC, Knight S, Balmaseda A, Harris E, et al. Analysis of Cross-Reactive Antibodies Recognizing the Fusion Loop of Envelope Protein and Correlation with Neutralizing Antibody Titers in Nicaraguan Dengue Cases. PLoS Negl Trop Dis [Internet]. 2013;7(9). Available from: http://www.embase.com/search/results?subaction=viewrecord&from=export&id=L369904074

70. Zompi S, Montoya M, Pohl MO, Balmaseda A, Harris E. Dominant cross-reactive B cell response during secondary acute dengue virus infection in humans. PLoS Negl Trop Dis [Internet]. 2012;6(3). Available from: http://www.embase.com/search/results?subaction=viewrecord&from=export&id=L364547191

71. Balakrishnan T, Bela-Ong DB, Toh YX, Flamand M, Devi S, Koh MB, et al. Dengue virus activates polyreactive, natural IgG B cells after primary and secondary infection. PLoS One [Internet]. 2011;6(12). Available from: http://www.embase.com/search/results?subaction=viewrecord&from=export&id=L363132805

72. Mansfield KL, Horton DL, Johnson N, Li L, Barrett ADT, Smith DJ, et al. Flavivirus-induced antibody cross-reactivity. J GEN VIROL [Internet]. 2011;92(12):2821–9. Available from: http://www.embase.com/search/results?subaction=viewrecord&from=export&id=L362939886

73. Blacksell SD, Jarman RG, Bailey MS, Tanganuchitcharnchai A, Jenjaroen K, Gibbons R V, et al. Evaluation of six commercial point-of-care tests for diagnosis of acute dengue infections: The need for combining NS1 antigen and IgM/IgG antibody detection to achieve acceptable levels of accuracy. Clin Vaccine Immunol [Internet]. 2011;18(12):2095–101. Available from: http://www.embase.com/search/results?subaction=viewrecord&from=export&id=L363085033

74. Litzba N, Klade CS, Lederer S, Niedrig M. Evaluation of serological diagnostic test systems assessing the immune response to Japanese encephalitis vaccination. PLoS Negl Trop Dis [Internet]. 2010;4(11). Available from: http://www.embase.com/search/results?subaction=viewrecord&from=export&id=L360094652

75. Moreno-Altamirano MMB, Sańchez-Garciá FJ, Loṕez-Martińez I, Rosales-Jimeńez C, Vaźquez-Pichardo M, Arriaga-Valona LJ, et al. Improving dengue virus diagnosis in rural areas of Mexico. Dengue Bull [Internet]. 2009;33(1):87–94. Available from: http://www.embase.com/search/results?subaction=viewrecord&from=export&id=L362968159

76. Crill WD, Hughes HR, Delorey MJ, Chang GJJ. Humoral immune responses of dengue fever patients using epitope-specific serotype-2 virus-like particle antigens. PLoS One [Internet]. 2009;4(4). Available from: http://www.embase.com/search/results?subaction=viewrecord&from=export&id=L354448146

77. Nuegoonpipat AA, Panthuyosri N, Anantapreecha S, Chanama S, Sa-Ngasang A, Sawanpanyalert P, et al. Cross-reactive IgM responses in patients with dengue or Japanese encephalitis. J Clin Virol [Internet]. 2008;42(1):75–7. Available from: http://www.embase.com/search/results?subaction=viewrecord&from=export&id=L50075103

78. Anantapreecha S, nuegoonpipat AA, Prakrong S, Chanama S, Sa-ngasang A, Sawanpanyalert P, et al. Dengue virus cross-reactive hemagglutination inhibition antibody responses in patients with primary dengue virus infection. Jpn J Infect Dis [Internet]. 2007;60(5):267–70. Available from: http://www.embase.com/search/results?subaction=viewrecord&from=export&id=L47516871

79. Vazquez S, Hafner G, Ruiz D, Calzada N, Guzman MG. Evaluation of immunoglobulin M and G capture enzyme-linked immunosorbent assay Panbio kits for diagnostic dengue infections. J Clin Virol [Internet]. 2007;39(3):194–8. Available from: http://www.embase.com/search/results?subaction=viewrecord&from=export&id=L46908966

80. Koraka P, Zeller H, Niedrig M, Osterhaus ADME, Groen J. Reactivity of serum samples from patients with a flavivirus infection measured by immunofluorescence assay and ELISA. Microbes Infect [Internet]. 2002;4(12):1209–15. Available from: http://www.embase.com/search/results?subaction=viewrecord&from=export&id=L35435274

81. Schwartz E, Mileguir F, Grossman Z, Mendelson E. Evaluation of ELISA-based sero-diagnosis of dengue fever in travelers. J Clin Virol [Internet]. 2000;19(3):169–73. Available from: http://www.embase.com/search/results?subaction=viewrecord&from=export&id=L30827217

82. McClain DJ, Pittman PR, Ramsburg HH, Nelson GO, Rossi CA, Mangiafico JA, et al. Immunologic interference from sequential administration of live attenuated alphavirus vaccines. J INFECT DIS [Internet]. 1998;177(3):634–41. Available from: http://www.embase.com/search/results?subaction=viewrecord&from=export&id=L28099877

83. Makino Y, Tadano M, Saito M, Maneekarn N, Sittisombut N, Sirisanthana V, et al. Studies on serological cross-reaction in sequential flavivirus infections. MICROBIOL IMMUNOL [Internet]. 1994;38(12):951–5. Available from: http://www.embase.com/search/results?subaction=viewrecord&from=export&id=L25002863

84. Chao DY, Whitney MT, Davis BS, Medina FA, Munoz JL, Chang GJJ. Comprehensive evaluation of differential serodiagnosis between Zika and dengue viral infections. J Clin Microbiol. 2019 Mar 1;57(3).

85. El Sahly HM, Gorchakov R, Lai L, Natrajan MS, Patel SM, Atmar RL, et al. Clinical, Virologic, and Immunologic Characteristics of Zika Virus Infection in a Cohort of US Patients: Prolonged RNA Detection in Whole Blood. Open forum infect dis [Internet]. 2019;6(1):ofy352. Available from: http://www.ncbi.nlm.nih.gov/entrez/query.fcgi?holding=inleurlib_fft&cmd=Retrieve&db=PubMed&dopt=Citation&list_uids=30697574

86. Pasquier C, Joguet G, Mengelle C, Chapuy-Regaud S, Pavili L, Prisant N, et al. Kinetics of anti-ZIKV antibodies after Zika infection using two commercial enzyme-linked immunoassays. Diagn Microbiol Infect Dis [Internet]. 2018;90(1):26–30. Available from: http://www.ncbi.nlm.nih.gov/entrez/query.fcgi?holding=inleurlib_fft&cmd=Retrieve&db=PubMed&dopt=Citation&list_uids=29107414

87. Schuller E, Klade CS, Heinz FX, Kollaritsch H, Rendi-Wagner P, Jilma B, et al. Effect of pre-existing anti-tick-borne encephalitis virus immunity on neutralising antibody response to the Vero cell-derived, inactivated Japanese encephalitis virus vaccine candidate IC51. Vaccine [Internet]. 2008;26(48):6151–6. Available from: http://www.ncbi.nlm.nih.gov/entrez/query.fcgi?holding=inleurlib_fft&cmd=Retrieve&db=PubMed&dopt=Citation&list_uids=18804132

88. Kuno G, Cropp CB, Wong-Lee J, Gubler DJ. Evaluation of an IgM immunoblot kit for dengue diagnosis. AM J TROP MED HYG [Internet]. 1998;59(5):757–62. Available from: http://www.ncbi.nlm.nih.gov/entrez/query.fcgi?holding=inleurlib_fft&cmd=Retrieve&db=PubMed&dopt=Citation&list_uids=9840593

89. Yu L, Wang RK, Gao F, Li M, Liu JY, Wang J, et al. Delineating antibody recognition against Zika virus during natural infection. JCI Insight. 2017;2(12).

90. Souza NCS e, Félix AC, de Paula AV, Levi JE, Pannuti CS, Romano CM. Evaluation of serological cross-reactivity between yellow fever and other flaviviruses. International Journal of Infectious Diseases [Internet]. 2019;81:4–5. Available from: http://www.sciencedirect.com/science/article/pii/S1201971219300347

91. Smith JL, Pugh CL, Cisney ED, Keasey SL, Guevara C, Ampuero JS, et al. Human Antibody Responses to Emerging Mayaro Virus and Cocirculating Alphavirus Infections Examined by Using Structural Proteins from Nine New and Old World Lineages. mSphere [Internet]. 2018;3(2):e00003-18. Available from: https://www.ncbi.nlm.nih.gov/pubmed/29577083

92. Portilho MM, Moraes LD, Kikuti M, Nascimento LCJ, Reis MG, Boaventura VS, et al. Accuracy of the Zika IgM antibody capture enzyme-linked immunosorbent assay from the centers for disease control and prevention (CDC Zika MAC-ELISA) for diagnosis of zika virus infection. Diagn [Internet]. 2020;10(10). Available from: https://www.embase.com/search/results?subaction=viewrecord&id=L2008368188&from=export

93. Ehmen C, Medialdea-Carrera R, Brown D, de Filippis AMB, de Sequeira P, Nogueira RMR, et al. Accurate detection of Zika virus IgG using a novel immune complex binding ELISA. TROP MED INT HEALTH [Internet]. 2021;26(1):89–101. Available from: https://www.embase.com/search/results?subaction=viewrecord&id=L2007397870&from=export

94. Echegaray F, Laing P, Hernandez S, Marquez S, Harris A, Laing I, et al. Adapting Rapid Diagnostic Tests to Detect Historical Dengue Virus Infections. Front Immunol [Internet]. 2021;12. Available from: https://www.embase.com/search/results?subaction=viewrecord&id=L635651193&from=export

95. Curren EJ, Venkat H, Sunenshine R, Fitzpatrick K, Kosoy O, Krow-Lucal E, et al. Assessment of Immunoglobulin M Enzyme-Linked Immunosorbent Assay Ratios to Identify West Nile Virus and St. Louis Encephalitis Virus Infections during Concurrent Outbreaks of West Nile Virus and St. Louis Encephalitis Virus Diseases, Arizona 2015. Vector Borne Zoonotic Dis [Internet]. 2020;20(8):619–23. Available from: https://www.embase.com/search/results?subaction=viewrecord&id=L632774877&from=export

96. Medina FA, Vila F, Premkumar L, Lorenzi O, Paz-Bailey G, Alvarado LI, et al. Capacity of a Multiplex IgM Antibody Capture ELISA to Differentiate Zika and Dengue Virus Infections in Areas of Concurrent Endemic Transmission. AM J TROP MED HYG [Internet]. 2022;106(2):585–92. Available from: https://www.embase.com/search/results?subaction=viewrecord&id=L2016866124&from=export

97. Pacenti M, Sinigaglia A, Martello T, de Rui ME, Franchin E, Pagni S, et al. Clinical and virological findings in patients with Usutu virus infection, northern Italy, 2018. Eurosurveillance [Internet]. 2019;24(47). Available from: https://www.embase.com/search/results?subaction=viewrecord&id=L2004835560&from=export

98. Sittikul P, Sriburin P, Rattanamahaphoom J, Limkittikul K, Sirivichayakul C, Chatchen S. Combining Immunoassays to Identify Zika Virus Infection in Dengue-Endemic Areas. Trop Med Infect Dis [Internet]. 2022;7(10). Available from: https://www.embase.com/search/results?subaction=viewrecord&id=L2019762263&from=export

99. Rojas A, Natrajan MS, Weber J, Cardozo F, Cantero C, Ananta JS, et al. Comparison of anti-dengue and anti-zika IgG on a plasmonic gold platform with neutralization testing. AM J TROP MED HYG [Internet]. 2021;104(5):1729–33. Available from: https://www.embase.com/search/results?subaction=viewrecord&id=L2011984338&from=export

100. Caracciolo I, Mora-Cardenas E, Aloise C, Carletti T, Segat L, Burali MS, et al. Comprehensive response to usutu virus following first isolation in blood donors in the friuli venezia giulia region of italy: Development of recombinant ns1-based serology and sensitivity to antiviral drugs. PLoS Negl Trop Dis [Internet]. 2020;14(3). Available from: https://www.embase.com/search/results?subaction=viewrecord&id=L2004180986&from=export

101. Barsosio HC, Gitonga JN, Karanja HK, Nyamwaya DK, Omuoyo DO, Kamau E, et al. Congenital microcephaly unrelated to flavivirus exposure in coastal Kenya. Wellcome Open Res [Internet]. 2019;4:179. Available from: http://dx.doi.org/10.12688/wellcomeopenres.15568.1

102. Lopes-Luz L, Junqueira IC, da Silveira LA, de Melo Pereira BR, da Silva LA, Ribeiro BM, et al. Dengue and Zika virus multi-epitope antigen expression in insect cells. Mol Biol Rep [Internet]. 2020;47(10):7333–40. Available from: https://www.embase.com/search/results?subaction=viewrecord&id=L2006841028&from=export

103. Auerswald H, Klepsch L, Schreiber S, Hülsemann J, Franzke K, Kann S, et al. The dengue ED3 dot assay, a novel serological test for the detection of denguevirus type-specific antibodies and its application in a retrospective seroprevalence study. Viruses [Internet]. 2019;11(4). Available from: https://www.embase.com/search/results?subaction=viewrecord&id=L2001795072&from=export

104. Pushpakumara PD, Jeewandara C, Gomes L, Perera Y, Wijewickrama A, Malavige GN, et al. Development and validation of an assay for detection of Japanese encephalitis virus specific antibody responses. PLoS One [Internet]. 2020;15(10 October). Available from: https://www.embase.com/search/results?subaction=viewrecord&id=L2008445543&from=export

105. Morales I, Rosenberger KD, Magalhaes T, Morais CNL, Braga C, Marques ETA, et al. Diagnostic performance of anti-zika virus igm, igam and igg elisas during co-circulation of zika, dengue, and chikungunya viruses in brazil and venezuela. PLoS Negl Trop Dis [Internet]. 2021;15(4). Available from: https://www.embase.com/search/results?subaction=viewrecord&id=L2007135181&from=export

106. Stiasny K, Malafa S, Aberle SW, Medits I, Tsouchnikas G, Aberle JH, et al. Different cross-reactivities of igm responses in dengue, zika and tick-borne encephalitis virus infections. Viruses [Internet]. 2021;13(4). Available from: https://www.embase.com/search/results?subaction=viewrecord&id=L2007134305&from=export

107. Falconi-Agapito F, Kerkhof K, Merino X, Michiels J, Van Esbroeck M, Bartholomeeusen K, et al. Dynamics of the Magnitude, Breadth and Depth of the Antibody Response at Epitope Level Following Dengue Infection. Front Immunol [Internet]. 2021;12. Available from: https://www.embase.com/search/results?subaction=viewrecord&id=L635504645&from=export

108. Yap TL, Hong SY, Soh JH, Ravichandraprabhu L, Lim VWX, Chan HM, et al. Engineered ns1 for sensitive, specifi c zika virus diagnosis from patient serology. Emerg Infect Dis [Internet]. 2021;27(5):1427–37. Available from: https://www.embase.com/search/results?subaction=viewrecord&id=L2011962526&from=export

109. Bouthry E, Hervé A, Brichler S, Poveda JD, Roque-Afonso AM, Vauloup-Fellous C. Evaluation and optimisation of commercial Zika IgG avidity assay. J Clin Virol [Internet]. 2020;124. Available from: https://www.embase.com/search/results?subaction=viewrecord&id=L2004846771&from=export

110. Martinez Viedma MDP, Panossian S, Gifford K, Garcia K, Figueroa I, Parham L, et al. Evaluation of ELISA-Based Multiplex Peptides for the Detection of Human Serum Antibodies Induced by Zika Virus Infection across Various Countries. 2021; Available from: http://dx.doi.org/10.3390/v13071319

111. Bonaparte M, Zheng L, Garg S, Guy B, Lustig Y, Schwartz E, et al. Evaluation of rapid diagnostic tests and conventional enzyme-linked immunosorbent assays to determine prior dengue infection. J Travel Med [Internet]. 2019;26(8). Available from: https://www.embase.com/search/results?subaction=viewrecord&id=L631315383&from=export

112. Loyola S, Huaman A, Popuche D, Castillo E, Ampuero JS, Silva M, et al. Evaluation of two serological assays for diagnosing zika virus infection. Diagn [Internet]. 2021;11(9). Available from: https://www.embase.com/search/results?subaction=viewrecord&id=L2014062009&from=export

113. Liao T, Wang X, Donolato M, Harris E, Cruz MM, Balmaseda A, et al. Evaluation of virotrack sero zika IgG/IgM, a new rapid and quantitative zika serological diagnostic assay. Diagn [Internet]. 2020;10(6). Available from: https://www.embase.com/search/results?subaction=viewrecord&id=L2006836778&from=export

114. Denis J, Attoumani S, Gravier P, Tenebray B, Garnier A, Briolant S, et al. High specificity and sensitivity of Zika EDIIIbased ELISA diagnosis highlighted by a large human reference panel. PLoS Negl Trop Dis [Internet]. 2019;13(9). Available from: https://www.embase.com/search/results?subaction=viewrecord&id=L2003458922&from=export

115. Tyson J, Tsai WY, Tsai JJ, Mässgård L, Stramer SL, Lehrer AT, et al. A high-throughput and multiplex microsphere immunoassay based on non-structural protein 1 can discriminate three flavivirus infections. PLoS Negl Trop Dis [Internet]. 2019;13(8). Available from: https://www.embase.com/search/results?subaction=viewrecord&id=L2003458864&from=export

116. Collins MH, Tu HA, Gimblet-Ochieng C, Liou GJA, Jadi RS, Metz SW, et al. Human antibody response to Zika targets type-specific quaternary structure epitopes. JCI Insight [Internet]. 2019;4(8). Available from: https://www.embase.com/search/results?subaction=viewrecord&id=L2001988817&from=export

117. Kareinen L, Hepojoki S, Huhtamo E, Korhonen EM, Schmidt-Chanasit J, Hedman K, et al. Immunoassay for serodiagnosis of Zika virus infection based on time-resolved Förster resonance energy transfer. PLoS One [Internet]. 2019;14(7). Available from: https://www.embase.com/search/results?subaction=viewrecord&id=L2002411681&from=export

118. Rodriguez-Barraquer I, Costa F, Nascimento EJM, Nery NJ, Castanha PMS, Sacramento GA, et al. Impact of preexisting dengue immunity on Zika virus emergence in a dengue endemic region. Science (1979) [Internet]. 2019;363(6427):607–10. Available from: http://dx.doi.org/10.1126/science.aav6618

119. Li X, Wan X, Liu J, Wang H, Li A, Ke C, et al. Luciferase Immunosorbent Assay Based on Multiple E Antigens for the Detection of Chikungunya Virus-Specific IgG Antibodies. Microbiol Spectr [Internet]. 2022;10(2). Available from: https://www.embase.com/search/results?subaction=viewrecord&id=L2017903653&from=export

120. Sharma M, Glasner DR, Watkins H, Puerta-Guardo H, Kassa Y, Egan MA, et al. Magnitude and Functionality of the NS1-Specific Antibody Response Elicited by a Live-Attenuated Tetravalent Dengue Vaccine Candidate. J INFECT DIS [Internet]. 2020;221(6):867–77. Available from: https://www.embase.com/search/results?subaction=viewrecord&id=L631295194&from=export

121. Tedder RS, Dicks S, Ijaz S, Santiago de Souza NC, Vincente de Paula A, Levy F, et al. Modulated Zika virus NS1 conjugate offers advantages for accurate detection of Zika virus specific antibody in double antigen binding and Ig capture enzyme immunoassays. PLoS One [Internet]. 2019;14(8). Available from: https://www.embase.com/search/results?subaction=viewrecord&id=L2002499096&from=export

122. Versiani AF, Martins EMN, Andrade LM, Cox L, Pereira GC, Barbosa-Stancioli EF, et al. Nanosensors based on LSPR are able to serologically differentiate dengue from Zika infections. Sci Rep [Internet]. 2020;10(1):11302. Available from: https://www.embase.com/search/results?subaction=viewrecord&id=L632319144&from=export

123. Amrun SN, Yee WX, Abu Bakar F, Lee B, Kam YW, Lum FM, et al. Novel differential linear B-cell epitopes to identify Zika and dengue virus infections in patients. Clin Transl Immunology [Internet]. 2019;8(7). Available from: https://www.embase.com/search/results?subaction=viewrecord&id=L628675667&from=export

124. Liberal V, Forrat R, Zhang C, Pan C, Bonaparte M, Yin W, et al. Performance Evaluation of a Dengue IgG Rapid Diagnostic Test Designed to Determine Dengue Serostatus as Part of Prevaccination Screening. Microbiol Spectr [Internet]. 2022;10(3). Available from: https://www.embase.com/search/results?subaction=viewrecord&id=L2019045577&from=export

125. Van der Beken Y, De Geyter D, Van Esbroeck M. Performance evaluation of the Diasorin LIAISON® XL Zika capture IgM CLIA test. Diagn Microbiol Infect Dis [Internet]. 2019;95(2):144–8. Available from: https://www.embase.com/search/results?subaction=viewrecord&id=L2002055860&from=export

126. Kizu JG, Graham M, Grant R, McCallum F, McPherson B, Auliff A, et al. Prevalence of Barmah Forest Virus, Chikungunya Virus and Ross River Virus Antibodies among Papua New Guinea Military Personnel before 2019 †. Viruses [Internet]. 2023;15(2). Available from: https://www.embase.com/search/results?subaction=viewrecord&id=L2021807845&from=export

127. Nunes JGC, Nunes BTD, Shan C, Moraes AF, Silva TR, de Mendonça MHR, et al. Reporter virus neutralization test evaluation for dengue and zika virus diagnosis in flavivirus endemic area. Pathogens [Internet]. 2021;10(7). Available from: https://www.embase.com/search/results?subaction=viewrecord&id=L2007811099&from=export

128. Gouel-Cheron A, Lumbard K, Hunsberger S, Arteaga-Cabello FJ, Beigel J, Belaunzarán-Zamudio PF, et al. Serial real-time RT-PCR and serology measurements substantially improve Zika and Dengue virus infection classification in a co-circulation area. Antiviral Res [Internet]. 2019;172. Available from: https://www.embase.com/search/results?subaction=viewrecord&id=L2003805847&from=export

129. Wongsawat J, Suttha P, Chanama S, Srisopa S, Yonchoho N, Limpanadusadee P. Serological differences after acute zika virus infections between children and adults: Implication for use of a serological test. AM J TROP MED HYG [Internet]. 2021;105(4):931–5. Available from: https://www.embase.com/search/results?subaction=viewrecord&id=L2015096300&from=export

130. Biggs JR, Sy AK, Brady OJ, Kucharski AJ, Funk S, Tu YH, et al. Serological evidence of widespread zika transmission across the Philippines. Viruses [Internet]. 2021;13(8). Available from: https://www.embase.com/search/results?subaction=viewrecord&id=L2013134954&from=export

131. Biggs JR, Sy AK, Brady OJ, Kucharski AJ, Funk S, Reyes MAJ, et al. A serological framework to investigate acute primary and post-primary dengue cases reporting across the Philippines. BMC Med [Internet]. 2020;18(1). Available from: https://www.embase.com/search/results?subaction=viewrecord&id=L2007391216&from=export

132. Zaidi MB, Cedillo-Barron L, González y Almeida ME, Garcia-Cordero J, Campos FD, Namorado-Tonix K, et al. Serological tests reveal significant cross-reactive human antibody responses to Zika and Dengue viruses in the Mexican population. Acta Trop. 2020 Jan 1;201:105201.

133. Calvert AE, Horiuchi K, Boroughs KL, Ong YT, Anderson KM, Biggerstaff BJ, et al. The Specificity of the Persistent IgM Neutralizing Antibody Response in Zika Virus Infections among Individuals with Prior Dengue Virus Exposure. J CLIN MICROBIOL [Internet]. 2021;59(8). Available from: https://www.embase.com/search/results?subaction=viewrecord&id=L2022955099&from=export

134. Densathaporn T, Sangthong R, Sakolnapa M, Surasombatpattana S, Kemapunmanus M, Masrinoul P, et al. Survey on neutralizing antibodies against Zika virus eighteen months post-outbreak in two southern Thailand communities. BMC Infect Dis [Internet]. 2020;20(1):921. Available from: http://dx.doi.org/10.1186/s12879-020-05654-8

135. Medialdea-Carrera R, Levy F, Castanha P, de Sequeira PC, Brasil P, Lewis-Ximenez LL, et al. A Systematic Evaluation of IgM and IgG Antibody Assay Accuracy in Diagnosing Acute Zika Virus Infection in Brazil: Lessons Relevant to Emerging Infections. J CLIN MICROBIOL [Internet]. 2021;59(12). Available from: https://www.embase.com/search/results?subaction=viewrecord&id=L2022955131&from=export

136. Shin A, Tukhanova N, Ndenkeh J, Shapiyeva Z, Yegemberdiyeva R, Yeraliyeva L, et al. Tick-borne encephalitis virus and West-Nile fever virus as causes of serous meningitis of unknown origin in Kazakhstan. Zoonoses Public Health [Internet]. 2022;69(5):514–25. Available from: https://www.embase.com/search/results?subaction=viewrecord&id=L2015397273&from=export

137. Girl P, Bestehorn-Willmann M, Zange S, Borde JP, Dobler G, von Buttlar H. Tick-borne encephalitis virus nonstructural protein 1 IgG enzyme-linked immunosorbent assay for differentiating infection versus vaccination antibody responses. J CLIN MICROBIOL [Internet]. 2020;58(4). Available from: https://www.embase.com/search/results?subaction=viewrecord&id=L2005438378&from=export

138. Zaaijer HL, Slot E, Molier M, Reusken CBEM, Koppelman MHGM. Usutu virus infection in Dutch blood donors. Transfusion (Paris) [Internet]. 2019;59(9):2931–7. Available from: https://www.embase.com/search/results?subaction=viewrecord&id=L628468130&from=export

139. Percivalle E, Cassaniti I, Sarasini A, Rovida F, Adzasehoun KMG, Colombini I, et al. West nile or usutu virus? A three-year follow-up of humoral and cellular response in a group of asymptomatic blood donors. Viruses [Internet]. 2020;12(2). Available from: https://www.embase.com/search/results?subaction=viewrecord&id=L2004829062&from=export

140. Langerak T, Kasbergen LMR, Chandler F, Brinkman T, Faerber Z, Phalai K, et al. Zika virus antibody titers three years after confirmed infection. Viruses [Internet]. 2021;13(7). Available from: https://www.embase.com/search/results?subaction=viewrecord&id=L2007878565&from=export

141. Katzelnick LC, Narvaez C, Arguello S, Mercado BL, Collado D, Ampie O, et al. Zika virus infection enhances future risk of severe dengue disease. Science (1979) [Internet]. 2020 Aug 28 [cited 2020 Nov 3];369(6507):1123–8. Available from: http://science.sciencemag.org/

142. Kam YW, Leite JA, Amrun SN, Lum FM, Yee WX, Bakar FA, et al. ZIKV-Specific NS1 Epitopes as Serological Markers of Acute Zika Virus Infection. J INFECT DIS [Internet]. 2019;220(2):203–12. Available from: https://www.embase.com/search/results?subaction=viewrecord&id=L629251081&from=export
